# Hospitalizations for COVID-19 Among US People Experiencing Incarceration or Homelessness

**DOI:** 10.1001/jamanetworkopen.2021.43407

**Published:** 2022-01-13

**Authors:** Martha P. Montgomery, Kai Hong, Kristie E. N. Clarke, Samantha Williams, Rena Fukunaga, Victoria L. Fields, Joohyun Park, Lyna Z. Schieber, Lyudmyla Kompaniyets, Colleen M. Ray, Lauren A. Lambert, Ashley S. D’Inverno, Tapas K. Ray, Alexiss Jeffers, Emily Mosites

**Affiliations:** 1 COVID-19 Emergency Response, Centers for Disease Control and Prevention, Atlanta, Georgia

## Abstract

**Importance:**

People experiencing incarceration (PEI) and people experiencing homelessness (PEH) have an increased risk of COVID-19 exposure from congregate living, but data on their hospitalization course compared with that of the general population are limited.

**Objective:**

To compare COVID-19 hospitalizations for PEI and PEH with hospitalizations among the general population.

**Design, Setting, and Participants:**

This cross-sectional analysis used data from the Premier Healthcare Database on 3415 PEI and 9434 PEH who were evaluated in the emergency department or were hospitalized in more than 800 US hospitals for COVID-19 from April 1, 2020, to June 30, 2021.

**Exposures:**

Incarceration or homelessness.

**Main Outcomes and Measures:**

Hospitalization proportions were calculated. and outcomes (intensive care unit admission, invasive mechanical ventilation [IMV], mortality, length of stay, and readmissions) among PEI and PEH were compared with outcomes for all patients with COVID-19 (not PEI or PEH). Multivariable regression was used to adjust for potential confounders.

**Results:**

In total, 3415 PEI (2952 men [86.4%]; mean [SD] age, 50.8 [15.7] years) and 9434 PEH (6776 men [71.8%]; mean [SD] age, 50.1 [14.5] years) were evaluated in the emergency department for COVID-19 and were hospitalized more often (2170 of 3415 [63.5%] PEI; 6088 of 9434 [64.5%] PEH) than the general population (624 470 of 1 257 250 [49.7%]) (*P* < .001). Both PEI and PEH hospitalized for COVID-19 were more likely to be younger, male, and non-Hispanic Black than the general population. Hospitalized PEI had a higher frequency of IMV (410 [18.9%]; adjusted risk ratio [aRR], 1.16; 95% CI, 1.04-1.30) and mortality (308 [14.2%]; aRR, 1.28; 95% CI, 1.11-1.47) than the general population (IMV, 88 897 [14.2%]; mortality, 84 725 [13.6%]). Hospitalized PEH had a lower frequency of IMV (606 [10.0%]; aRR, 0.64; 95% CI, 0.58-0.70) and mortality (330 [5.4%]; aRR, 0.53; 95% CI, 0.47-0.59) than the general population. Both PEI and PEH had longer mean (SD) lengths of stay (PEI, 9 [10] days; PEH, 11 [26] days) and a higher frequency of readmission (PEI, 128 [5.9%]; PEH, 519 [8.5%]) than the general population (mean [SD] length of stay, 8 [10] days; readmission, 28 493 [4.6%]).

**Conclusions and Relevance:**

In this cross-sectional study, a higher frequency of COVID-19 hospitalizations for PEI and PEH underscored the importance of adhering to recommended prevention measures. Expanding medical respite may reduce hospitalizations in these disproportionately affected populations.

## Key Points

**Question:**

How do COVID-19 hospitalizations for people experiencing incarceration or homelessness compare with those among the general US population?

**Findings:**

In a cross-sectional study using hospital discharge records from more than 800 hospitals, people experiencing incarceration who were evaluated in the emergency department had a higher frequency of hospitalization, invasive mechanical ventilation, mortality, and readmissions, as well as longer lengths of stay, compared with the general population. People experiencing homelessness who were evaluated in the emergency department had a higher frequency of hospitalization and readmissions, a lower frequency of invasive mechanical ventilation and mortality, and longer lengths of stay compared with the general population.

**Meaning:**

This study suggests that expanding medical respite may reduce hospitalizations or shorten the length of stay for COVID-19 for people experiencing incarceration or homelessness who are disproportionately affected by the pandemic.

## Teaser

This cross-sectional study uses data from more than 800 hospitals to compare COVID-19 hospitalizations for people experiencing incarceration or homelessness with COVID-19 hospitalizations among the general population.

## Introduction

People experiencing incarceration (PEI) and people experiencing homelessness (PEH) often live in congregate settings where large outbreaks of SARS-CoV-2 can occur rapidly.^1,2^ Many PEI and PEH are at increased risk for severe illness from COVID-19 because of underlying medical conditions.^3,4^ An estimated 2.1 million people are incarcerated nationally, with approximately two-thirds in state and federal prisons (typically people serving sentences of >1 year) and one-third in local jails and detention centers (typically detained for <1 year).^5^ On a given night, there are an estimated 580 000 PEH in the US, with approximately 6 in 10 staying in sheltered locations.^6^ Assessing COVID-19 illness severity and health care use, including hospitalizations, length of stay, and readmissions, is essential to understanding the disease burden for PEI and PEH.

Both populations experience barriers to accessing health care. For PEI, the government is required to provide health care; decisions on when and how to hospitalize patients vary by facility and jurisdiction.^7^ Many PEH lack regular health care, have competing priorities (eg, housing, food, or employment), or experience financial or transportation difficulties.^8^ These barriers could lead to increased hospitalizations or more severe outcomes if diagnosis and treatment of COVID-19 are delayed.

Gaps remain in understanding COVID-19 hospitalizations for PEI and PEH. Previous reports were isolated to a single state, were limited in sample size, or were unable to adjust for potential confounding demographic factors.^9,10,11^ In this report, we examine COVID-19 emergency department visits and hospitalizations among PEI and PEH compared with the general population using a national electronic health record database.

## Methods

### Data Source and Participants

In this cross-sectional study, we analyzed data from the Premier Healthcare Database Special COVID-19 Release (release version 09/28/2021), an all-payer, hospital-based administrative database that contains hospital discharge records from more than 800 for-profit and nonprofit, community and teaching hospitals across the United States; the database is updated every 2 weeks.^12^ We included all adults aged 18 years or older with COVID-19 who were evaluated in the emergency department or hospitalized and discharged during the period from April 1, 2020, through June 30, 2021. COVID-19 was defined using the *International Statistical Classification of Diseases and Related Health Problems, Tenth Revision, Clinical Modification* (*ICD-10-CM*) code U07.1 listed as either the primary or secondary diagnosis code.^13^ PEI and PEH were identified using *ICD-10-CM* codes listed as either the primary or secondary diagnosis code during any emergency department visit or hospitalization during the period from April 1, 2020, through June 30, 2021 (Table 1). People experiencing incarceration were also identified using the admission code “admitted from court/law enforcement.” The discharge code for court or law enforcement was not included in the PEI definition to avoid selection bias toward patients alive at discharge. Because no standardized method for identifying PEI from hospital discharge records exists, we examined results separately for PEI identified by *ICD-10-CM* codes and PEI identified by admission code as a sensitivity analysis. Patients coded as both PEI and PEH were included in the PEI sample because we considered PEH who are incarcerated to be housed. The general population comparison group included all adults with COVID-19 who were not identified as PEI or PEH. Patients with unknown sex and those discharged to court or law enforcement were excluded. This activity was reviewed by the Centers for Disease Control and Prevention (CDC) and was conducted consistent with applicable federal law and CDC policy (45 CFR part 46). This study was exempt from institutional review board oversight and exempt from patient informed consent because the disclosed Premier Healthcare Database Special COVID-19 Release data are considered deidentified. This report followed the Strengthening the Reporting of Observational Studies in Epidemiology (STROBE) reporting guideline for cross-sectional studies.^14^

**Table 1.** Inclusion Criteria for PEI and PEH

Code	No. (%)^a^
PEI (n = 3415)^b^	
Code Z65.1 (imprisonment and other incarceration)	1162 (34.0)
Codes Y92.140-Y92.149 (prison as the place of occurrence of the external cause)	228 (6.7)
Admission code (admitted from court or law enforcement)	2565 (75.1)
PEH cohort (n = 9434)^b^	
Code Z59.0 (homelessness)	8773 (93.0)
Code Z59.1 (inadequate housing)	81 (0.9)
Code Z59.8 (other problems related to housing and economic circumstances)	300 (3.2)
Code Z59.9 (problem related to housing and economic circumstances, unspecified)	401 (4.3)

Abbreviations: *ICD-10-CM*, *International Statistical Classification of Diseases and Related Health Problems, Tenth Revision, Clinical Modification*; PEH, people experiencing homelessness; PEI, people experiencing incarceration.

^a^ Individual *ICD-10-CM* codes and admission codes sum to more than the total because patients could have more than 1 code.

^b^ Patients with codes for both populations are included in the PEI cohort.

### Measures

We defined hospitalization proportion as the number of patients hospitalized for COVID-19 out of the total number evaluated in the emergency department for COVID-19. Patient race and ethnicity were determined as recorded in the electronic health record. Underlying medical conditions were defined using *ICD-10-CM* codes listed as a primary or secondary diagnosis code during any inpatient or outpatient encounter during the period from January 1, 2019, through the initial COVID-19 encounter (eTable 1 in the Supplement). We included underlying medical conditions defined in a previous analysis with modifications to align with a CDC list of medical conditions associated with severe illness for COVID-19.^15,16^ We examined 2 additional medical categories that disproportionately affect PEI and PEH but are not included in the CDC list: serious mental illness (eg, severe major depression or schizophrenia) and disability (eg, intellectual, developmental, or physical disability).

Among hospitalized patients, we examined several outcomes: acute in-hospital complications, laboratory test results, intensive care unit admission, invasive mechanical ventilation (IMV), in-hospital mortality, length of stay, and 30-day readmission for COVID-19. We identified 7 laboratory test results associated with severe outcomes in COVID-19 based on meta-analyses: leukocytosis, lymphocytopenia, and elevated d-dimer, C-reactive protein, lactate dehydrogenase, aspartate aminotransferase, and alanine aminotransferase levels.^17,18^ We examined the proportion of patients with laboratory abnormalities above or below the normal reference range as defined by each facility. Acute in-hospital complications (eg, respiratory failure and acute kidney failure) were defined using *ICD-10-CM* diagnosis or procedure codes listed as a primary or secondary diagnosis code during the same COVID-19 hospitalization (eTable 2 in the Supplement).^19^

### Statistical Analysis

We examined frequencies of demographic characteristics and underlying medical conditions and conducted Pearson χ^2^ tests (or Fisher exact tests for cell sizes <5) to determine whether PEI and PEH had the same frequencies as the general population.^20^ We then calculated intensive care unit admission, IMV, in-hospital mortality, length of stay, and 30-day readmission for COVID-19 for PEI and PEH compared with the general population using multivariable regression analyses. We obtained risk ratios using either a log binomial model (intensive care unit admission and IMV) or an alternative revised Poisson model when the log binomial model did not converge (in-hospital mortality and readmission).^21^ We used a zero-truncated negative binomial model for length of stay, which was an overdispersed positive count data variable.^22^

For the regression models, we calculated unadjusted, age-adjusted, and fully adjusted models. In the fully adjusted model, we adjusted for age, sex (male or female), race and ethnicity (non-Hispanic Asian, non-Hispanic Black, Hispanic, non-Hispanic White, non-Hispanic other race and ethnicity [unspecified non-Hispanic race and ethnicity categories that have been suppressed owing to small sample size and confidentiality], or unknown race and ethnicity), health care professional region (Northeast, Midwest, South, or West), health care professional urbanicity (rural or urban), pandemic wave (first, second, or third), serious mental illness (yes or no), and disability status (yes or no), which were selected based on a priori understanding of the direction of causality. Payer source (Medicare, Medicaid, private insurance, self-pay, or other payer source) and underlying medical conditions were not included in the final model because these factors are more likely predicated on incarceration and housing status rather than potential confounders. We accounted for clustering at the hospital level by calculating 95% CIs based on clustered SEs in log binomial models and revised Poisson models or by including a hospital random effect in zero-truncated negative binomial models. SAS software, version 9.4 (SAS Institute Inc) was used to conduct all statistical analyses. All *P* values were from 2-sided tests, and results were deemed statistically significant at *P* < .05.

## Results

The analysis included discharge records from 892 hospitals. We identified 3415 PEI (2952 men [86.4%]; mean [SD] age, 50.8 [15.7] years), 9434 PEH (6776 men [71.8%]; mean [SD] age, 50.1 [14.5] years), and 1 257 250 patients in the general population with COVID-19 who were evaluated in the emergency department only or hospitalized (Table 2). The proportion of hospitalized patients was higher for PEI (2170 [63.5%]; *P* < .001) and PEH (6088 [64.5%]; *P* < .001) than the general population (624 470 [49.7%]). PEI and PEH evaluated only in the emergency department for COVID-19 were more likely to be male (1013 of 1245 PEI [81.4%]; 2349 of 3346 PEH [70.2%]; *P* < .001) and non-Hispanic Black (315 of 1245 PEI [25.3%]; 923 of 3346 PEH [27.6%]; *P* < .001) and less likely to be non-Hispanic Asian (13 of 1245 PEI [1.0%]; 37 of 3346 [1.1%] PEH; *P* < .001) and of Hispanic ethnicity (193 of 1245 [15.5%] PEI; 435 of 3346 [13.0%] PEH; *P* < .001) than the general population (male, 274 952 of 632 780 [43.5%]; non-Hispanic Black, 131 230 of 632 780 [20.7%]; non-Hispanic Asian, 13 717 of 632 780 [2.2%]; Hispanic, 140 110 of 632 780 [22.1%]). Several underlying medical conditions were more common among PEI and PEH with COVID-19 in the emergency department than among the general population, including chronic obstructive pulmonary disease, liver disease, tobacco use, substance use disorder, and serious mental illness.

**Table 2.** Demographic Characteristics and Medical Conditions for PEI and PEH With COVID-19 in the US, April 2020 to June 2021

Characteristic	Emergency department only					Hospitalized				
	PEI (n = 1245)^a^		General population (n = 632 780), No. (%)	PEH (n = 3346)^a^		PEI (n = 2170)^a^		General population (n = 624 470), No. (%)	PEH (n = 6088)^a^	
	No. (%)	*P* value^b^		No. (%)	*P* value^b^	No. (%)	*P* value^b^		No. (%)	*P* value^b^
Hospitalization proportion, No./total No. (%)	NA	NA	NA	NA	NA	2170/3415 (63.5)	<.001	624 470/1 257 250 (49.7)	6088/9434 (64.5)	<.001
Age, y										
Median (IQR)	42 (31-55)	NA	47 (33-61)	46 (34-57)	NA	56 (44-65)	NA	65 (52-77)	55 (43-63)	NA
<25	120 (9.6)	.61	63 741 (10.1)	212 (6.3)	<.001	40 (1.8)	.34	13 386 (2.1)	176 (2.9)	<.001
25-44	576 (46.3)	<.001	225 557 (35.7)	1387 (41.5)	<.001	508 (23.4)	<.001	85 138 (13.6)	1564 (25.7)	<.001
45-54	233 (18.7)	.71	115 823 (18.3)	732 (21.9)	<.001	447 (20.6)	<.001	80 118 (12.8)	1298 (21.3)	<.001
55-64	182 (14.6)	.10	103 473 (16.4)	730 (21.8)	<.001	582 (26.8)	<.001	123 180 (19.7)	1849 (30.4)	<.001
65-74	95 (7.6)	<.001	69 270 (11.0)	246 (7.4)	<.001	420 (19.4)	.001	139 204 (22.3)	908 (14.9)	<.001
≥75	39 (3.1)	<.001	54 916 (8.7)	39 (1.2)	<.001	173 (8.0)	<.001	183 444 (29.4)	293 (4.8)	<.001
Sex										
Male	1013 (81.4)	<.001	274 952 (43.5)	2349 (70.2)	<.001	1939 (89.4)	<.001	318 510 (51.0)	4427 (72.7)	<.001
Female	232 (18.6)	<.001	357 828 (56.6)	997 (29.8)	<.001	231 (10.7)	<.001	305 960 (49.0)	1661 (27.3)	<.001
Race and ethnicity										
Non-Hispanic Asian	13 (1.0)	.007	13 717 (2.2)	37 (1.1)	<.001	22 (1.0)	<.001	16 179 (2.6)	87 (1.4)	<.001
Non-Hispanic Black	315 (25.3)	<.001	131 230 (20.7)	923 (27.6)	<.001	613 (28.3)	<.001	111 245 (17.8)	1494 (24.5)	<.001
Hispanic	193 (15.5)	<.001	140 110 (22.1)	435 (13.0)	<.001	292 (13.5)	<.001	103 639 (16.6)	853 (14.0)	<.001
Non-Hispanic White	560 (45.0)	.32	293 503 (46.4)	1611 (48.2)	.04	978 (45.1)	<.001	339 147 (54.3)	2940 (48.3)	<.001
Non-Hispanic other race^c^	144 (11.6)	<.001	52 565 (8.3)	329 (9.8)	.001	200 (9.2)	.32	53 803 (8.6)	674 (11.1)	<.001
Unknown	33 (2.7)	.61	15 372 (2.4)	48 (1.4)	<.001	87 (4.0)	<.001	16 636 (2.7)	127 (2.1)	.005
Geographical divisions^d^										
Northeast	100 (8.0)	.01	64 847 (10.3)	353 (10.6)	.57	143 (6.6)	<.001	112 411 (18.0)	1030 (16.9)	.03
Midwest	304 (24.4)	.01	135 659 (21.4)	569 (17.0)	<.001	528 (24.3)	<.001	130 982 (21.0)	812 (13.3)	<.001
South	453 (36.4)	<.001	331 793 (52.4)	1061 (31.7)	<.001	932 (43.0)	<.001	292 644 (46.9)	2355 (38.7)	<.001
West	388 (31.2)	<.001	100 481 (15.9)	1363 (40.7)	<.001	567 (26.1)	<.001	88 433 (14.2)	1891 (31.1)	<.001
Rural vs urban health care location^d^										
Rural	400 (32.1)	<.001	98 510 (15.6)	231 (6.9)	<.001	431 (19.9)	<.001	75 581 (12.1)	356 (5.9)	<.001
Urban	845 (67.9)	<.001	534 270 (84.4)	3115 (93.1)	<.001	1739 (80.1)	<.001	548 889 (87.9)	5732 (94.2)	<.001
Payer source^d^										
Medicare	89 (7.2)	<.001	138 792 (21.9)	680 (20.3)	.03	192 (8.9)	<.001	332 021 (53.2)	1847 (30.3)	<.001
Medicaid	288 (23.1)	.06	132 648 (21.0)	1991 (59.5)	<.001	768 (35.4)	<.001	84 615 (13.6)	2963 (48.7)	<.001
Private insurance	262 (21.0)	<.001	255 608 (40.4)	183 (5.5)	<.001	415 (19.1)	<.001	157 986 (25.3)	414 (6.8)	<.001
Self-pay	90 (7.2)	.95	45 450 (7.2)	264 (7.9)	.11	46 (2.1)	.04	17 820 (2.9)	505 (8.3)	<.001
Other	516 (41.5)	<.001	60 282 (9.5)	228 (6.8)	<.001	749 (34.5)	<.001	32 028 (5.2)	359 (5.9)	.007
Underlying medical conditions^e^										
Asthma	116 (9.3)	.52	55 702 (8.8)	496 (14.8)	<.001	195 (9.0)	.32	60 082 (9.6)	790 (13.0)	<.001
COPD	100 (8.0)	<.001	35 751 (5.7)	503 (15.0)	<.001	424 (19.5)	.005	107 615 (17.2)	1432 (23.5)	<.001
Cystic fibrosis	0	>.99	62 (0.01)	0	>.99	0	>.99	118 (0.02)	<10	NA
Pulmonary fibrosis	<10	NA	2060 (0.3)	15 (0.5)	.21	39 (1.8)	>.99	11 227 (1.8)	71 (1.2)	<.001
Other lung conditions	38 (3.1)	.006	12 400 (2.0)	250 (7.5)	<.001	128 (5.9)	<.001	52 145 (8.4)	835 (13.7)	<.001
Heart disease	162 (13.0)	.12	73 286 (11.6)	695 (20.8)	<.001	582 (26.8)	<.001	246 183 (39.4)	2392 (39.3)	.83
Hypertension	359 (28.8)	.62	178 399 (28.2)	1192 (35.6)	<.001	1060 (48.9)	.22	313 324 (50.2)	2773 (45.6)	<.001
Sickle cell and thalassemia	<10	NA	634 (0.1)	<10	NA	<10	NA	1207 (0.2)	14 (0.2)	.52
Cancer	20 (1.6)	.61	11 390 (1.8)	53 (1.6)	.35	106 (4.9)	<.001	41 658 (6.7)	286 (4.7)	<.001
Cerebrovascular diseases	17 (1.4)	.63	7699 (1.2)	89 (2.7)	<.001	69 (3.2)	<.001	32 863 (5.3)	312 (5.1)	.63
Neurologic or musculoskeletal	156 (12.5)	<.001	43 678 (6.9)	743 (22.2)	<.001	570 (26.3)	<.001	202 062 (32.4)	2137 (35.1)	<.001
Down syndrome	0	>.99	312 (0.1)	<10	NA	0	.02	1361 (0.2)	<10	NA
Diabetes	182 (14.6)	.21	100 765 (15.9)	722 (21.6)	<.001	789 (36.4)	<.001	263 921 (42.3)	2233 (36.7)	<.001
Overweight	14 (1.1)	.40	5695 (0.9)	77 (2.3)	<.001	75 (3.5)	.02	28 423 (4.6)	274 (4.5)	.85
Obesity	75 (6.0)	.14	44 877 (7.1)	342 (10.2)	<.001	335 (15.4)	<.001	128 356 (20.6)	1003 (16.5)	<.001
Severe obesity	37 (3.0)	.05	25 739 (4.1)	185 (5.5)	<.001	213 (9.8)	<.001	97 861 (15.7)	719 (11.8)	<.001
Liver diseases	60 (4.8)	<.001	18 933 (3.0)	391 (11.7)	<.001	292 (13.5)	<.001	55 607 (8.9)	1385 (22.8)	<.001
Chronic kidney disease, including dialysis	60 (4.8)	.33	34 469 (5.5)	275 (8.2)	<.001	392 (18.1)	<.001	165 601 (26.5)	1393 (22.9)	<.001
Immunosuppression	67 (5.4)	.10	27 890 (4.4)	372 (11.1)	<.001	324 (14.9)	.08	102 009 (16.3)	1134 (18.6)	<.001
Substance use disorder	243 (19.5)	<.001	19 487 (3.1)	1767 (52.8)	<.001	342 (15.8)	<.001	36 023 (5.8)	3480 (57.2)	<.001
Tobacco use	554 (44.5)	<.001	143 178 (22.6)	2310 (69.0)	<.001	1239 (57.1)	<.001	222 836 (35.7)	4197 (68.9)	<.001
Underlying medical condition listed above										
None	365 (29.3)	<.001	267 012 (42.2)	325 (9.7)	<.001	135 (6.2)	.02	46 832 (7.5)	134 (2.2)	<.001
Any 1	308 (24.7)	.57	152 128 (24.0)	556 (16.6)	<.001	266 (12.3)	.31	72 177 (11.6)	405 (6.7)	<.001
Any 2	225 (18.1)	<.001	90 593 (14.3)	699 (20.9)	<.001	421 (19.4)	<.001	102 029 (16.3)	865 (14.2)	<.001
≥3	347 (27.9)	<.001	123 047 (19.5)	1766 (52.8)	<.001	1348 (62.1)	.02	403 432 (64.6)	4684 (76.9)	<.001
Other medical conditions of interest^e^										
Serious mental illness	156 (12.5)	<.001	12 433 (2.0)	1082 (32.3)	<.001	264 (12.2)	<.001	24 588 (3.9)	1712 (28.1)	<.001
Disability	57 (4.6)	.04	22 202 (3.5)	302 (9.0)	<.001	182 (8.4)	<.001	85 755 (13.7)	929 (15.3)	<.001
Wave										
Wave 1 (April-May 2020)	85 (6.8)	.18	37 532 (5.9)	274 (8.2)	<.001	293 (13.5)	<.001	68 796 (11.0)	844 (13.9)	<.001
Wave 2 (June-August 2020)	227 (18.2)	.93	114 793 (18.1)	538 (16.1)	.002	355 (16.4)	.14	95 027 (15.2)	1107 (18.2)	<.001
Wave 3 (September 2020 to June 2021)	933 (74.9)	.42	480 455 (75.9)	2534 (75.7)	.79	1522 (70.1)	<.001	460 647 (73.8)	4137 (68.0)	<.001

Abbreviations: COPD, chronic obstructive pulmonary disease; *ICD-10-CM*, *International Statistical Classification of Diseases and Related Health Problems, Tenth Revision, Clinical Modification*; NA, not applicable; PEH, people experiencing homelessness; PEI, people experiencing incarceration.

^a^ Patients with *ICD-10-CM* codes for both categories are included in the PEI cohort (77 for emergency department; 187 for hospitalization).

^b^ Compared with the general population.

^c^ Including other unspecified non-Hispanic race categories that have been suppressed owing to small sample size and confidentiality.

^d^ Categories are mutually exclusive based on the first hospitalization for COVID-19.

^e^ Categories are not mutually exclusive. Underlying medical conditions and other medical conditions of interest were defined using *ICD-10-CM* codes listed as a primary or secondary diagnosis code during any inpatient or outpatient encounter from January 1, 2019, through the initial COVID-19 encounter (eTable 1 in the Supplement).

PEI and PEH hospitalized with COVID-19 were more likely to be younger (median age: PEI, 56 years [IQR, 44-65 years]; PEH, 55 years [IQR, 43-63]; general population, 65 years [IQR, 52-77 years]), male (PEI, 1939 [89.4%]; PEH, 4427 [72.7%]; general population, 318 510 [51.0%]), and non-Hispanic Black (PEI, 613 [28.3%]; PEH, 1494 [24.5%]; general population, 111 245 [17.8%]) and less likely to be non-Hispanic Asian (PEI, 22 [1.0%]; PEH, 87 [1.4%]; general population, 16 179 [2.6%]), non-Hispanic White (PEI, 978 [45.1%]; PEH, 2940 [48.3%]; general population, 339 147 [54.3%]), and of Hispanic ethnicity (PEI, 292 [13.5%]; PEH, 853 [14.0%]; general population, 103 639 [16.6%]) than the general population (Table 2). The health care location was more likely to be rural for hospitalized PEI (431 [19.9%]) than for the general population (75 581 [12.1%]; *P* < .001), whereas the health care location was more likely to be urban for PEH (5732 [94.2%]) than for the general population (548 889 [87.9%]; *P* < .001). Despite their younger age, there were fewer PEI (135 [6.2%]; *P* = .02) and PEH (134 [2.2%]; *P* < .001) hospitalized for COVID-19 with no underlying medical conditions compared with the general population (46 832 [7.5%]). Significant differences were seen for individual conditions. For example, the proportion of individuals hospitalized for COVID-19 with severe obesity was lower for PEI (213 [9.8%]; *P* < .001) and PEH (719 [11.8%]; *P* < .001) than for the general population (97 861 [15.7%]), whereas liver diseases were more frequent for PEI (292 [13.5%]; *P* < .001) and PEH (1385 [22.8%]; *P* < .001) than for the general population (55 607 [8.9%]). Serious mental illness was higher among PEI (264 [12.2%]; *P* < .001) and PEH (1712 [28.1%]; *P* < .001) than among the general population (24 588 [3.9%]).

The overall frequency of in-hospital complications was lower for hospitalized PEI (1812 [83.5%]) and PEH (4400 [72.3%]) than for the general population (561 322 [89.9%]) (Table 3). Two respiratory conditions, pneumonia and respiratory failure, were more frequent among the general population (pneumonia, 487 806 [78.1%]; respiratory failure, 347 798 [55.7%]) than among PEI (pneumonia, 1522 [70.1%]; respiratory failure, 1134 [52.3%]) or PEH (pneumonia, 3085 [50.7%]; respiratory failure, 1958 [32.2%]). Individual complications with higher frequency for PEI compared with the general population included acute respiratory distress syndrome (183 [8.4%] vs 44 155 [7.1%]) and acute hepatitis or liver failure (53 [2.4%] vs 8709 [1.4%]). For PEH, most complications (eg, respiratory, renal, and sepsis) were less frequent than among the general population, with a few exceptions. A higher proportion of PEH than patients in the general population experienced acute congestive heart failure (468 [7.7%] vs 32 867 [5.3%]), hypertensive crisis (237 [3.9%] vs 11 633 [1.9%]), and diabetic ketoacidosis (175 of 2233 [7.8%] vs 10 912/263 921 [4.1%]), despite the younger age distribution. Laboratory test results were unavailable for most hospitals. Among 263 of 860 hospitals (30.6%) with available data, 3 laboratory test result abnormalities (white blood cell count, C-reactive protein, and alanine aminotransferase) were significantly more frequent and 2 laboratory test result abnormalities (d-dimer and lactate dehydrogenase) were less frequent among PEI than among the general population. All 7 laboratory test result abnormalities were significantly less frequent among PEH than among the general population.

**Table 3.** In-Hospital Complications and Laboratory Values for PEI and PEH Hospitalized for COVID-19, April 2020 to June 2021

Characteristic	PEI (n = 2170)^a^		General population (n = 624 470), No. (%)	PEH (n = 6088)^a^	
	No. (%)	Difference (95% CI)^b^		No. (%)	Difference (95% CI)^b^
In-hospital complications					
Any complication	1812 (83.5)	−6.4 (−8.0 to −4.8)	561 322 (89.9)	4400 (72.3)	−17.6 (−18.7 to −16.5)
Respiratory	1629 (75.1)	−8.0 (−9.9 to −6.2)	518 889 (83.1)	3514 (57.7)	−25.4 (−26.6 to −24.1)
Pneumonia	1522 (70.1)	−8.0 (−9.9 to −6.1)	487 806 (78.1)	3085 (50.7)	−27.4 (−28.7 to −26.2)
Respiratory failure	1134 (52.3)	−3.4 (−5.5 to −1.3)	347 798 (55.7)	1958 (32.2)	−23.5 (−24.7 to −22.4)
ARDS	183 (8.4)	1.4 (0.2 to 2.5)	44 155 (7.1)	184 (3.0)	−4.1 (−4.5 to −3.6)
COPD exacerbation, No./total No. (%)^c^	80/424 (18.9)	−0.6 (−4.3 to 3.1)	20 958/10 7615 (19.5)	315/1432 (22.0)	2.5 (0.4 to 4.7)
Cardiac	250 (11.5)	−1.5 (−2.9 to −0.2)	81 579 (13.1)	936 (15.4)	2.3 (1.4 to 3.2)
Acute myocardial infarction or unstable angina	171 (7.9)	0.4 (−0.7 to 1.6)	46 561 (7.5)	402 (6.6)	−0.9 (−1.5 to −0.2)
Acute congestive heart failure	74 (3.4)	−1.9 (−2.6 to −1.1)	32 867 (5.3)	468 (7.7)	2.4 (1.8 to 3.1)
Hypertensive crisis	33 (1.5)	−0.3 (−0.9 to 0.2)	11 633 (1.9)	237 (3.9)	2.0 (1.5 to 2.5)
Hematologic or vascular	133 (6.1)	−0.2 (−1.2 to 0.8)	39 474 (6.3)	363 (6.0)	−0.4 (−1.0 to 0.2)
Neurologic	48 (2.2)	−0.2 (−0.8 to 0.5)	14 842 (2.4)	142 (2.3)	−0.0 (−0.4 to 0.3)
Cerebral ischemia or infarction	41 (1.9)	0.1 (−0.5 to 0.6)	11 429 (1.8)	104 (1.7)	−0.1 (−0.5 to 0.2)
Endocrine	52 (2.4)	−0.1 (−0.7 to 0.6)	15 304 (2.5)	223 (3.7)	1.2 (0.7 to 1.7)
Diabetic ketoacidosis, No./total No. (%)^c^	44/789 (5.6)	1.4 (−0.2 to 3.1)	10 912/263 921 (4.1)	175/2233 (7.8)	3.7 (2.6 to 4.8)
Gastrointestinal	67 (3.1)	1.1 (0.4 to 1.8)	12 519 (2.0)	154 (2.5)	0.5 (0.1 to 0.9)
Acute hepatitis or liver failure	53 (2.4)	1.1 (0.4 to 1.7)	8709 (1.4)	84 (1.4)	−0.0 (−0.3 to 0.3)
Renal	657 (30.3)	−1.3 (−3.2 to 0.7)	196 943 (31.5)	1594 (26.2)	−5.4 (−6.5 to −4.2)
Acute kidney failure	632 (29.1)	−0.9 (−2.8 to 1.0)	187 471 (30.0)	1519 (25.0)	−5.1 (−6.2 to −4.0)
Dialysis initiation	59 (2.7)	−0.1 (−0.8 to 0.6)	17 403 (2.8)	127 (2.1)	−0.7 (−1.1 to −0.3)
Sepsis	556 (25.6)	−1.6 (−3.4 to 0.3)	169 956 (27.2)	1351 (22.2)	−5.0 (−6.1 to −4.0)
Laboratory values					
Elevated white blood cell count	267 (12.3)	1.8 (0.4 to 3.2)	65 553 (10.5)	358 (5.9)	−4.6 (−5.2 to −4.0)
Decreased lymphocyte count	379 (17.5)	0.8 (−0.8 to 2.4)	104 258 (16.7)	612 (10.1)	−6.6 (−7.4 to −5.9)
Elevated d-dimer	191 (8.8)	−2.6 (−3.8 to −1.4)	70 906 (11.4)	337 (5.5)	−5.8 (−6.4 to −5.2)
Elevated C-reactive protein	308 (14.2)	2.4 (0.9 to 3.8)	73 874 (11.8)	358 (5.9)	−6.0 (−6.6 to −5.4)
Elevated lactate dehydrogenase	171 (7.9)	−1.9 (−3.0 to −0.7)	60 845 (9.7)	252 (4.1)	−5.6 (−6.1 to −5.1)
Elevated AST	262 (12.1)	1.3 (−0.1 to 2.7)	67 196 (10.8)	394 (6.5)	−4.3 (−4.9 to −3.7)
Elevated ALT	196 (9.0)	1.9 (0.7 to 3.1)	44 557 (7.1)	255 (4.2)	−3.0 (−3.5 to −2.4)

Abbreviations: ALT, alanine aminotransferase; ARDS, acute respiratory distress syndrome; AST, aspartate aminotransferase; COPD, chronic obstructive pulmonary disease; *ICD-10-CM*, *International Statistical Classification of Diseases and Related Health Problems, Tenth Revision, Clinical Modification*; PEH, people experiencing homelessness; PEI, people experiencing incarceration.

^a^ Patients with *ICD-10-CM* codes for both categories are included in the PEI cohort (n = 187).

^b^ Difference between the general population.

^c^ Proportion calculated using the denominator of individuals with the condition identified as an underlying medical condition.

PEI hospitalized for COVID-19 were more likely than the general population to require IMV (410 [18.9%] vs 88 897 [14.2%]; adjusted risk ratio [aRR], 1.16; 95% CI, 1.04-1.30) and experience in-hospital mortality (308 [14.2%] vs 84 725 [13.6%]; aRR, 1.28; 95% CI, 1.11-1.47) than the general population after adjusting for age and other covariates (Table 4). PEH hospitalized for COVID-19 had a lower frequency of IMV (606 [10.0%]; aRR, 0.64; 95% CI, 0.58-0.70) and in-hospital mortality (330 [5.4%]; aRR, 0.53; 95% CI, 0.47-0.59) than the general population. Intensive care unit admission was not significantly different for PEI or PEH compared with the general population. Fully adjusted estimates for all covariates are included in eTable 3 in the Supplement. In sensitivity analyses, results for PEI identified by *ICD-10-CM* codes alone were similar (eTable 4 in the Supplement). Results for PEI identified by admission code alone found no difference in IMV or mortality compared with the general population.

**Table 4.** Unadjusted and Adjusted Hospitalization Outcomes for PEI and PEH With COVID-19, April 2020 to June 2021

Outcome	PEI (n = 2170)^a^				General population (n = 624 470), No. (%)	PEH (n = 6088)^a^			
	No. (%)	RR (95% CI)^b^				No. (%)	RR (95% CI)^b^		
		Unadjusted	Age-adjusted	Fully adjusted^c^			Unadjusted	Age-adjusted	Fully adjusted^c^
Hospitalization outcomes									
Intensive care unit admission	683 (31.5)	0.99 (0.80-1.23)	1.04 (0.84-1.29)	0.95 (0.80-1.13)	197 683 (31.7)	1922 (31.6)	1.00 (0.90-1.10)	1.05 (0.96-1.16)	0.92 (0.85-1.01)
Invasive mechanical ventilation	410 (18.9)	1.33 (1.19-1.48)	1.39 (1.25-1.55)	1.16 (1.04-1.30)	88 897 (14.2)	606 (10.0)	0.70 (0.63-0.77)	0.74 (0.67-0.82)	0.64 (0.58-0.70)
In-hospital mortality	308 (14.2)	1.05 (0.89-1.23)	1.47 (1.28-1.69)	1.28 (1.11-1.47)	84 725 (13.6)	330 (5.4)	0.40 (0.35-0.45)	0.61 (0.54-0.69)	0.53 (0.47-0.59)
30-d Readmission for COVID-19	128 (5.9)	1.29 (1.06-1.57)	1.55 (1.26-1.89)	1.45 (1.18-1.78)	28 493 (4.6)	519 (8.5)	1.87 (1.71-2.04)	2.34 (2.14-2.55)	2.10 (1.92-2.30)
Length of stay, mean (SD), d	9 (10)	1.11 (1.06-1.16)^d^	1.19 (1.14-1.24)^d^	1.11 (1.06-1.16)^d^	8 (10)	11 (26)	1.23 (1.20-1.26)^d^	1.33 (1.29-1.36)^d^	1.24 (1.20-1.27)^d^

Abbreviations: *ICD-10-CM*, *International Statistical Classification of Diseases and Related Health Problems, Tenth Revision, Clinical Modification*; PEH, people experiencing homelessness; PEI, people experiencing incarceration; RR, risk ratio.

^a^ Patients with *ICD-10-CM* codes for both categories are included in the PEI cohort (n = 187).

^b^ For intensive care and invasive mechanical ventilation, RRs were obtained from log-binomial models. For mortality and readmission, RRs were obtained from an alternative revised Poisson model.

^c^ Covariates included age (10-year increment), sex, race and ethnicity, geographic divisions, rural/urban, serious mental illness, and disability.

^d^ Incidence rate ratios (95% CIs) were obtained from a zero-truncated negative binomial model.

Readmission for COVID-19 within 30 days of hospital discharge was more common for PEI (128 [5.9%]; aRR, 1.45; 95% CI, 1.18-1.78) and PEH (519 [8.5%]; aRR, 2.10; 95% CI, 1.92-2.30) than for the general population (28 493 [4.6%]) (Table 4). The mean (SD) length of stay was longer for PEI (9 [10] days; incidence rate ratio, 1.11; 95% CI, 1.06-1.16) and PEH (11 [26] days; incidence rate ratio, 1.24; 95% CI, 1.20-1.27) than for the general population (8 [10] days).

## Discussion

This analysis expands on previous reports of COVID-19 hospitalization among PEI and PEH by using a large database of geographically diverse US hospitals and including a general population group for comparison. We found that PEI and PEH with COVID-19 who were evaluated in the emergency department were hospitalized more often, had longer lengths of stay, and had more frequent readmission than the general population. PEI hospitalized for COVID-19 had increased IMV and mortality compared with the general population. PEH hospitalized with COVID-19 had a less severe hospital course. Because we do not have population denominators for PEI or PEH, we cannot draw conclusions about population-level COVID-19 risk or severity compared with the general population. Previous population-based estimates have found that COVID-19 case rates and COVID-19 mortality rates were higher among PEI and PEH compared with the general population.^23,24^ In contrast, we calculated in-hospital measures using hospital admissions as the denominator; therefore, interpreting our findings requires understanding factors associated with both illness severity and reasons for hospitalization.

Our finding of increased IMV among PEI hospitalized for COVID-19 compared with the general population is consistent with an earlier publication.^10^ Our findings additionally identified a higher proportion of PEI hospitalized with a longer length of stay and more frequent readmission. There are several possible explanations for why PEI referred to the emergency department are hospitalized with more severe illness. Some correctional facilities can provide basic medical treatment on site, which could reduce the need for hospitalization, particularly for less severe cases of COVID-19.^7,25^ The decision to transport an individual to a hospital is often made by a corrections medical director or contracted health care agency based on established preapproval processes.^7^ Correctional agencies must consider the costs and potential exposures for staff to provide transportation and in-hospital supervision. These factors could select for hospitalization of individuals with more severe cases. Individual PEI might perceive medical isolation space within correctional facilities as a form of punishment, akin to solitary confinement, which could lead to delayed presentation for illness. Facilities and clinicians should consider the institutional restrictions defined for this setting and ensure that these institutional barriers do not interfere with providing appropriate levels of health care to PEI.^26^ Several factors beyond illness severity may also be associated with longer duration of hospitalization for PEI. Hospitalizations might be extended if a correctional facility lacks medical rehabilitation space, equipment, or staffing to provide recuperative care at discharge or if delays occur in arranging staff to provide return transportation.

Our findings of severe COVID-19 illness among PEI underscore the importance of following recommended prevention measures. Because of overcrowding and limited availability of resources (eg, staffing, space, and health care), correctional and detention centers have been urged to consider COVID-19–related risks when making bail decisions.^27^ Reducing jail and prison populations has allowed some facilities to provide the necessary medical isolation and quarantine spaces and has allowed for as much physical distancing as possible. Actions to prevent the spread of SARS-CoV-2 within the facility and between the community and the facility have been critical in lowering infection risk. Recommended infection, prevention, and control strategies include incorporating physical distancing and masking; reinforcing hygiene practices; intensifying facility cleaning and disinfection; conducting symptom and temperature screening for staff, visitors, and PEI; testing symptomatic and asymptomatic individuals; establishing appropriate medical isolation and quarantine cohorting; and offering vaccinations to staff and PEI.^28^ Strengthening partnerships between health departments and correctional facilities and agencies can help to effectively implement these infection, prevention, and control strategies.

For PEH, we found that individuals evaluated in the emergency department were admitted with COVID-19 more often but were less likely to require IMV and less likely to die compared with the general population, consistent with an earlier report from a smaller, single-center study.^11^ There are at least 3 possible explanations for these findings. First, COVID-19 could exacerbate underlying conditions that lead to a higher frequency of hospitalization but not necessarily to a higher frequency of IMV or mortality for PEH compared with the general population. This possibility is supported by our finding that complications, such as acute congestive heart failure, hypertensive crisis, and diabetic ketoacidosis, were more common among PEH, whereas COVID-19 complications, such as respiratory and kidney complications, were more common in the general population. PEH with inadequate access to routine primary care may have undiagnosed or poorly controlled underlying medical conditions that are exacerbated by COVID-19. Second, PEH with asymptomatic COVID-19 might receive a diagnosis of COVID-19 during the workup for other conditions more often than the general population given the high use of the emergency department among this population.^29,30^ Third, PEH might be hospitalized for reasons associated with their housing status, such as an inability to recuperate or self-isolate while infectious. The inability to recuperate or self-isolate in a safe location might also lead to discharge delays and longer lengths of stay, as has been observed for PEH with other medical conditions.^31^

Communities have developed solutions to address the lack of safe recuperation and isolation space. Medical respite care provides a safe location for PEH to recover from illness and offers medical and social services. A systematic review found that medical respite for PEH was associated with reduced future hospital admissions, lengths of stay, and readmissions.^32^ More recent studies have demonstrated the cost-effectiveness of medical respite programs.^33,34^ Expanding the availability of medical respite programs for PEH during the COVID-19 pandemic may have long-lasting benefits for PEH.

### Limitations

This report has some limitations. The main limitation is our ability to identify PEI and PEH from *ICD-10-CM* and admission codes. Our findings might not be generalizable to all PEI and PEH hospitalized for COVID-19, and *ICD-10-CM* codes are likely insufficient to identify all hospitalized PEI.^35,36^ We included admission codes in our PEI definition to broaden the scope, which provided more conservative estimates than *ICD-10-CM* codes alone. For PEH, *ICD-10-CM* codes are a more established method of identification but are underused, and hospitals might not have standardized methods for recording housing status in electronic health records. It remains unclear whether PEH identified through *ICD-10-CM* codes are generalizable to the broader population of hospitalized PEH.^37,38,39,40^ The *ICD-10-CM* codes for homelessness could be used preferentially for individuals who are admitted or who cannot be discharged for reasons associated with their housing status, which could bias our estimates away from the null. However, the importance of medical respite care for these populations remains. In addition, we were unable to deduplicate individuals who accessed care from multiple hospital systems; however, we expect that this small number would be offset by the large sample size. We relied on *ICD-10-CM* diagnoses to identify obesity and other underlying medical conditions, which likely resulted in an underestimation, but we expect that the underestimation would be similar for all 3 populations. Most hospitals did not report laboratory test results; however, the low reporting is consistent for all 3 populations, there was adequate sample size for analysis, and the findings are consistent with other outcomes (eg, IMV and mortality). Last, although payer status and underlying medical conditions were not included in the fully adjusted model, we conducted sensitivity analyses that included these variables separately and found no meaningful changes in our results (eTable 4 in the Supplement).

## Conclusions

In this cross-sectional study, PEI and PEH who presented to the emergency department with COVID-19 were hospitalized more often than the general population. Increased lengths of stay and readmission rates highlight the complex factors outside of COVID-19 illness with which PEI and PEH must contend and support the expansion of medical respite facilities. The high rates of COVID-19 hospitalizations among PEI and PEH reinforce the importance of COVID-19 prevention measures for these disproportionately affected populations. In the long term, reducing COVID-19 hospitalizations among PEI and PEH will require continued partnerships among homeless services, correctional facilities and agencies, health care professionals, and public health agencies to ensure that COVID-19 vaccinations and other prevention measures are implemented equitably for PEI and PEH.

## Notes

Supplement. **eTable 1.** List of *ICD-10-CM* and Procedure Codes for Underlying Medical Conditions **eTable 2.** List of *ICD-10-CM* and Procedure Codes for Acute Complications **eTable 3.** Full Multivariable Results for COVID-19 Hospitalization Outcomes Among People Experiencing Incarceration and People Experiencing Homelessness, United States, April 2020–June 2021 **eTable 4.** Sensitivity Analyses for COVID-19 Hospitalization Outcomes for People Experiencing Incarceration and People Experiencing Homelessness, United States, April 2020–June 2021
