# Supplementary material for: Hospitalizations for COVID-19 Among US People Experiencing Incarceration or Homelessness
Source: JAMA Netw Open. 2022 Jan 13;5(1):e2143407. doi: 10.1001/jamanetworkopen.2021.43407 (PMC8759002; doi:10.1001/jamanetworkopen.2021.43407)
Supplement: Supplement. — eTable 1. List of ICD-10-CM and Procedure Codes for Underlying Medical Conditions eTable 2. List of ICD-10-CM and Procedure Codes for Acute Complications eTable 3. Full Multivariable Results for COVID-19 Hospitalization Outcomes Among People Experiencing Incarceration and People Experiencing Homelessness, United States, April 2020–June 2021 eTable 4. Sensitivity Analyses for COVID-19 Hospitalization Outcomes for People Experiencing Incarceration and People Experiencing Homelessness, United States, April 2020–June 2021 [file jamanetwopen-e2143407-s001.pdf]

Supplementary Online Content

Montgomery MP, Hong K, Clarke KEN, et al. Hospitalizations for COVID-19 among US people experiencing incarceration or homelessness. *JAMA Netw Open.* 2022;5(1):e2143407. doi:10.1001/jamanetworkopen.2021.43407

eTable 1. List of *ICD-10-CM* and Procedure Codes for Underlying Medical Conditions
eTable 2. List of *ICD-10-CM* and Procedure Codes for Acute Complications
eTable 3. Full Multivariable Results for COVID-19 Hospitalization Outcomes Among People Experiencing Incarceration and People Experiencing Homelessness, United States, April 2020–June 2021
eTable 4. Sensitivity Analyses for COVID-19 Hospitalization Outcomes for People Experiencing Incarceration and People Experiencing Homelessness, United States, April 2020–June 2021

This supplementary material has been provided by the authors to give readers additional information about their work.

© 2022 Montgomery MP et al. *JAMA Network Open.*

eTable 1. List of *ICD-10-CM* and Procedure Codes for Underlying Medical Conditions

**Asthma**	
Moderate persistent asthma	J45.4
Severe persistent asthma	J45.5
Other and unspecified asthma	J45.9
Other asthma	J45.99
**Chronic obstructive pulmonary disease (COPD)**	
Bronchitis, not specified as acute or chronic	J40
Simple and mucopurulent chronic bronchitis	J41
Unspecified chronic bronchitis	J42
Emphysema	J43
Other chronic obstructive pulmonary disease	J44
**Cystic fibrosis**	
Cystic fibrosis	E84
**Pulmonary fibrosis**	
Other interstitial pulmonary diseases	J84
**Other lung conditions**	
Respiratory tuberculosis	A15
Pulmonary mycobacterial infection	A31.0
Histoplasmosis	B39
Blastomycosis	B40
Paracoccidioimycosis	B41
Aspergillosis	B44
Cryptococcosis	B45
Pulmonary mucormycosis	B46.0
Sarcoidosis of lung	D86.0
Alpha-1-antitrypsin deficiency	E88.01
Bronchiectasis	J47
Coalworker's pneumoconiosis	J60
Pneumoconiosis due to asbestos and other mineral fibers	J61
Pneumoconiosis due to dust containing silica	J62
Pneumoconiosis due to other inorganic dusts	J63
Unspecified pneumoconiosis	J64
Pneumoconiosis associated with tuberculosis	J65

© 2022 Montgomery MP et al. *JAMA Network Open.*

eTable 1. List of *ICD-10-CM* and Procedure Codes for Underlying Medical Conditions

Airway disease due to specific organic dust	J66
Hypersensitivity pneumonitis due to organic dust	J67
Respiratory conditions due to inhalation of chemicals, gases, fumes and vapors	J68
Pneumonitis due to solids and liquids*	J69
Respiratory conditions due to other external agents	J70
Pulmonary edema*	J81
Pulmonary eosinophilia, not elsewhere classified	J82
Tracheostomy complications*	J95.0
Respiratory failure, not elsewhere classified*	J96
Pulmonary collapse*	J98.1
Respiratory disorders in diseases classified elsewhere	J99
Interstitial emphysema and related conditions originating in the perinatal period	P25
Pulmonary hemorrhage originating in perinatal period	P26
Chronic respiratory disease originating in the perinatal period	P27
Other respiratory conditions originating in perinatal period	P28
Congenital malformations of lung	Q33
Complications of heart-lung transplant	T86.3
Complications of lung transplant	T86.8
Lung transplant status	Z94.2
**Heart disease**	
Hypertensive heart disease	I11
Hypertensive heart and chronic kidney disease	I13
ST elevation (STEMI) and non-ST elevation (NSTEMI) myocardial infarction*	I21
Subsequent ST elevation (STEMI) and non-ST elevation (NSTEMI) myocardial infarction	I22
Certain current complications following ST elevation (STEMI) and non-ST elevation (NSTEMI) myocardial infarction (within the 28 day period)	I23
Other acute ischemic heart diseases*	I24
Chronic ischemic heart disease	I25
Other pulmonary heart diseases	I27
Cardiomyopathy	I42
Cardiomyopathy in diseases classified elsewhere	I43
Cardiac arrest*	I46
Heart transplant status	Z94.1

© 2022 Montgomery MP et al. *JAMA Network Open.*

eTable 1. List of *ICD-10-CM* and Procedure Codes for Underlying Medical Conditions

Presence of cardiac and vascular implants and grafts	Z95
Coronary angioplasty status	Z98.61
Heart failure	I50
Rheumatic fever with heart involvement	I01
Rheumatic chorea	I02
Rheumatic mitral valve diseases	I05
Rheumatic aortic valve diseases	I06
Rheumatic tricuspid valve diseases	I07
Multiple valve diseases	I08
Other rheumatic heart diseases	I09
Pulmonary embolism*	I26
Other diseases of pulmonary vessels	I28
Other diseases of pericardium	I31
Nonrheumatic mitral valve disorders	I34
Nonrheumatic aortic valve disorders	I35
Nonrheumatic tricuspid valve disorders	I36
Nonrheumatic pulmonary valve disorders	I37
Myocarditis in diseases classified elsewhere	I41
Atrioventricular and left bundle-branch block*	I44
Atrial fibrillation and flutter*	I48
Complications and ill-defined descriptions of heart disease	I51
Other heart disorders in diseases classified elsewhere	I52
Aortic aneurysm and dissection*	I71
Other aneurysm*	I72
Other peripheral vascular diseases	I73
Arterial embolism and thrombosis*	I74
Atheroembolism*	I75
Disorders of arteries, arterioles and capillaries in diseases classified elsewhere	I79
Postcardiotomy syndrome	I97.0
Other postprocedural cardiac functional disturbances	I97.1
Other necrotizing vasculopathies	M31
Congenital malformations of cardiac chambers and connections	Q20
Congenital malformations of cardiac septa	Q21

© 2022 Montgomery MP et al. *JAMA Network Open.*

eTable 1. List of *ICD-10-CM* and Procedure Codes for Underlying Medical Conditions

Congenital malformations of pulmonary and tricuspid valves	Q22
Congenital malformations of aortic and mitral valves	Q23
Other congenital malformations of heart	Q24
Congenital malformations of great arteries	Q25
Congenital malformations of great veins	Q26
Congenital absence and hypoplasia of umbilical artery	Q27.0
Arteriovenous malformation (peripheral)	Q27.3
Congenital phlebectasia	Q27.4
Other specified congenital malformations of peripheral vascular system	Q27.8
Congenital malformation of peripheral vascular system, unspecified	Q27.9
Other congenital malformations of circulatory system	Q28
Situs inversus	Q89.3
**Hypertension**	
Essential (primary) hypertension	I10
**Sickle cell and thalassemia**	
Alpha thalassemia	D56.0
Beta thalassemia	D56.1
Delta-beta thalassemia	D56.2
Hemoglobin E-beta thalassemia	D56.5
Thalassemia, unspecified	D56.9
Hb-SS disease with crisis	D57.0
Sickle-cell disease without crisis	D57.1
Sickle-cell/Hb-C disease	D57.2
Sickle-cell thalassemia	D57.4
Other sickle-cell disorders	D57.8
**Cancer**	
Malignant neoplasm of lip	C00
Malignant neoplasm of base of tongue	C01
Malignant neoplasm of other and unspecified parts of tongue	C02
Malignant neoplasm of gum	C03
Malignant neoplasm of floor of mouth	C04
Malignant neoplasm of palate	C05
Malignant neoplasm of other and unspecified parts of mouth	C06

© 2022 Montgomery MP et al. *JAMA Network Open.*

eTable 1. List of *ICD-10-CM* and Procedure Codes for Underlying Medical Conditions

Malignant neoplasm of parotid gland	C07
Malignant neoplasm of other and unspecified major salivary glands	C08
Malignant neoplasm of tonsil	C09
Malignant neoplasm of oropharynx	C10
Malignant neoplasm of nasopharynx	C11
Malignant neoplasm of pyriform sinus	C12
Malignant neoplasm of hypopharynx	C13
Malignant neoplasm of other and ill-defined sites in the lip, oral cavity and pharynx	C14
Malignant neoplasm of esophagus	C15
Malignant neoplasm of stomach	C16
Malignant neoplasm of small intestine	C17
Malignant neoplasm of colon	C18
Malignant neoplasm of rectosigmoid junction	C19
Malignant neoplasm of rectum	C20
Malignant neoplasm of anus and anal canal	C21
Malignant neoplasm of liver and intrahepatic bile ducts	C22
Malignant neoplasm of gallbladder	C23
Malignant neoplasm of other and unspecified parts of biliary tract	C24
Malignant neoplasm of pancreas	C25
Malignant neoplasm of other and ill-defined digestive organs	C26
Malignant neoplasm of nasal cavity and middle ear	C30
Malignant neoplasm of accessory sinuses	C31
Malignant neoplasm of larynx	C32
Malignant neoplasm of trachea	C33
Malignant neoplasm of bronchus and lung	C34
Malignant neoplasm of thymus	C37
Malignant neoplasm of heart, mediastinum and pleura	C38
Malignant neoplasm of other and ill-defined sites in the respiratory system and intrathoracic organs	C39
Malignant neoplasm of bone and articular cartilage of limbs	C40
Malignant neoplasm of bone and articular cartilage of other and unspecified sites	C41
Malignant melanoma of skin	C43
Other and unspecified malignant neoplasm of skin	C44
Mesothelioma	C45

© 2022 Montgomery MP et al. *JAMA Network Open.*

eTable 1. List of *ICD-10-CM* and Procedure Codes for Underlying Medical Conditions

Kaposi's sarcoma	C46
Malignant neoplasm of peripheral nerves and autonomic nervous system	C47
Malignant neoplasm of retroperitoneum and peritoneum	C48
Malignant neoplasm of other connective and soft tissue	C49
Merkel cell carcinoma	C4A
Malignant neoplasms of breast	C50
Malignant neoplasm of vulva	C51
Malignant neoplasm of vagina	C52
Malignant neoplasm of cervix uteri	C53
Malignant neoplasm of corpus uteri	C54
Malignant neoplasm of uterus, part unspecified	C55
Malignant neoplasm of ovary	C56
Malignant neoplasm of other and unspecified female genital organs	C57
Malignant neoplasm of placenta	C58
Malignant neoplasm of penis	C60
Malignant neoplasm of prostate	C61
Malignant neoplasm of testis	C62
Malignant neoplasm of other and unspecified male genital organs	C63
Malignant neoplasm of kidney, except renal pelvis	C64
Malignant neoplasm of renal pelvis	C65
Malignant neoplasm of ureter	C66
Malignant neoplasm of bladder	C67
Malignant neoplasm of other and unspecified urinary organs	C68
Malignant neoplasm of eye and adnexa	C69
Malignant neoplasm of meninges	C70
Malignant neoplasm of brain	C71
Malignant neoplasm of spinal cord, cranial nerves and other parts of central nervous system	C72
Malignant neoplasm of thyroid gland	C73
Malignant neoplasm of adrenal gland	C74
Malignant neoplasm of other endocrine glands and related structures	C75
Malignant neoplasm of other and ill-defined sites	C76
Secondary and unspecified malignant neoplasm of lymph nodes	C77
Secondary malignant neoplasm of respiratory and digestive organs	C78

© 2022 Montgomery MP et al. *JAMA Network Open.*

eTable 1. List of *ICD-10-CM* and Procedure Codes for Underlying Medical Conditions

Secondary malignant neoplasm of other and unspecified sites	C79
Malignant neuroendocrine tumors	C7A
Secondary neuroendocrine tumors	C7B
Malignant neoplasm without specification of site	C80
Hodgkin lymphoma	C81
Follicular lymphoma	C82
Non-follicular lymphoma	C83
Mature T/NK-cell lymphomas	C84
Other specified and unspecified types of non-Hodgkin lymphoma	C85
Other specified types of T/NK-cell lymphoma	C86
Malignant immunoproliferative diseases and certain other B-cell lymphomas	C88
Multiple myeloma and malignant plasma cell neoplasms	C90
Lymphoid leukemia	C91
Myeloid leukemia	C92
Monocytic leukemia	C93
Other leukemias of specified cell type	C94
Leukemia of unspecified cell type	C95
Other and unspecified malignant neoplasms of lymphoid, hematopoietic and related tissue	C96
Melanoma in situ	D03
Myelodysplastic syndromes	D46
**Cerebrovascular diseases**	
Nontraumatic subarachnoid hemorrhage*	I60
Nontraumatic intracerebral hemorrhage*	I61
Other and unspecified nontraumatic intracranial hemorrhage*	I62
Cerebral infarction*	I63
Other cerebrovascular diseases	I67
Cerebrovascular disorders in diseases classified elsewhere	I68
Sequelae of cerebrovascular disease	I69
**Neurological/Musculoskeletal**	
Tay-Sachs disease	E75.02
Other gangliosidosis	E75.19
Neuronal ceroid lipofuscinosis	E75.4
Vascular dementia	F01

© 2022 Montgomery MP et al. *JAMA Network Open.*

eTable 1. List of *ICD-10-CM* and Procedure Codes for Underlying Medical Conditions

Dementia in other diseases classified elsewhere	F02
Unspecified dementia	F03
Moderate intellectual disabilities	F71
Severe intellectual disabilities	F72
Profound intellectual disabilities	F73
Rett's syndrome	F84.2
Huntington's disease	G10
Hereditary ataxia	G11
Spinal muscular atrophy and related syndromes	G12
Systemic atrophies primarily affecting central nervous system in diseases classified elsewhere	G13
Postpolio syndrome	G14
Parkinson's disease	G20
Secondary parkinsonism	G21
Other degenerative diseases of basal ganglia	G23
Dystonia	G24
Other extrapyramidal and movement disorders	G25
Extrapyramidal and movement disorders in diseases classified elsewhere	G26
Alzheimer's disease	G30
Other degenerative diseases of nervous system, not elsewhere classified	G31
Other degenerative disorders of nervous system in diseases classified elsewhere	G32
Multiple sclerosis	G35
Other acute disseminated demyelination	G36
Other demyelinating diseases of central nervous system	G37
Epilepsy and recurrent seizures	G40
Transient cerebral ischemic attacks and related syndromes*	G45
Vascular syndromes of brain in cerebrovascular diseases	G46
Hereditary and idiopathic neuropathy	G60
Inflammatory polyneuropathy	G61
Other and unspecified polyneuropathies	G62
Polyneuropathy in diseases classified elsewhere	G63
Other disorders of peripheral nervous system	G64
Myasthenia gravis and other myoneural disorders	G70
Primary disorders of muscles	G71

© 2022 Montgomery MP et al. *JAMA Network Open.*

eTable 1. List of *ICD-10-CM* and Procedure Codes for Underlying Medical Conditions

Disorders of myoneural junction and muscle in diseases classified elsewhere	G73
Cerebral palsy	G80
Hemiplegia and hemiparesis	G81
Paraplegia (paraparesis) and quadriplegia (quadriparesis)	G82
Other paralytic syndromes	G83
Multi-system degeneration of the autonomic nervous system	G90.3
Hydrocephalus	G91
Other disorders of brain	G93
Other disorders of brain in diseases classified elsewhere	G94
Other and unspecified diseases of spinal cord	G95
Myelopathy in diseases classified elsewhere	G99.2
Kearns-Sayre syndrome	H49.81
Chronic postrheumatic arthropathy (Jaccoud)	M12.0
Dermato(poly)myositis in neoplastic disease	M36.0
Other disturbances of cerebral status of newborn	P91
Anencephaly and similar malformations	Q00
Encephalocele	Q01
Microcephaly	Q02
Congenital hydrocephalus	Q03
Other congenital malformations of brain	Q04
Spina bifida	Q05
Other congenital malformations of the spinal cord	Q06
Other congenital malformations of nervous system	Q07
Congenital malformations of spine and bony thorax	Q76
Osteochondrodysplasia with defects of growth of tubular bones and spine	Q77
Other osteochondrodysplasias	Q78
Congenital malformations of musculoskeletal system, not elsewhere classified	Q79
Phakomatoses, not elsewhere classified	Q85
Marfan's syndrome	Q87.4
Trisomy 18 and Trisomy 13	Q91
Other trisomies and partial trisomies of the autosomes, not elsewhere classified	Q92
Monosomies and deletions from the autosomes, not elsewhere classified	Q93
Turner's syndrome	Q96

© 2022 Montgomery MP et al. *JAMA Network Open.*

eTable 1. List of *ICD-10-CM* and Procedure Codes for Underlying Medical Conditions

Other symptoms and signs involving cognitive functions and awareness	R41
Functional quadriplegia	R53.2
Age-related physical debility/frailty	R54
**Down syndrome**	
Down syndrome	Q90
**Diabetes**	
Diabetes mellitus due to underlying condition	E08
Type 1 diabetes mellitus	E10
Type 2 diabetes mellitus	E11
Other specified diabetes mellitus	E13
**Overweight**	
Overweight	E66.3
Body mass index (BMI) overweight, 25–29.9	Z68.25–Z68.29
**Obesity**	
Other obesity due to excess calories	E66.09
Drug-induced obesity	E66.1
Other obesity	E66.8
Body mass index (BMI) obese, 30–39	Z68.3
Obesity, unspecified	E66.9
**Severe obesity**	
Morbid (severe) obesity due to excess calories	E66.01
Morbid obesity with alveolar hypoventilation (pickwickian syndrome)	E66.2
Body mass index (BMI) 40 or greater, adult	Z68.4
**Liver diseases**	
Chronic viral hepatitis	B18
Portal vein thrombosis*	I81
Esophageal varices	I85
Alcoholic liver disease	K70
Toxic liver disease	K71
Hepatic failure, not elsewhere classified	K72
Chronic hepatitis, not elsewhere classified	K73
Fibrosis and cirrhosis of liver	K74
Other inflammatory liver diseases	K75

© 2022 Montgomery MP et al. *JAMA Network Open.*

eTable 1. List of *ICD-10-CM* and Procedure Codes for Underlying Medical Conditions

Other diseases of liver	K76
Liver disorders in diseases classified elsewhere	K77
**Chronic kidney disease including dialysis**	
Hypertensive chronic kidney disease	I12
Chronic kidney disease	N18
Encounter for care involving renal dialysis	Z49
Patient's noncompliance with renal dialysis	Z91.15
Kidney transplant status	Z94.0
Dependence on renal dialysis	Z99.2
Rapidly progressive nephritic syndrome	N01
Recurrent and persistent hematuria	N02
Chronic nephritic syndrome	N03
Nephrotic syndrome	N04
Unspecified nephritic syndrome	N05
Isolated proteinuria with specified morphological lesion	N06
Hereditary nephropathy, not elsewhere defined	N07
Glomerular disorders in diseases classified elsewhere	N08
Chronic tubulo-interstitial nephritis	N11
Drug- and heavy-metal-induced tubulo-interstitial and tubular conditions	N14
Other renal tubulo-interstitial diseases	N15
Renal tubulo-interstitial disorders in diseases classified elsewhere	N16
Disorders resulting from impaired renal tubular function	N25
Unspecified contracted kidney	N26
Other disorders of kidney and ureter, not elsewhere classified	N28
Congenital renal artery stenosis	Q27.1
Other congenital malformations of renal artery	Q27.2
Renal agenesis and other reduction defects of kidney	Q60
**Immunosuppression**	
Human immunodeficiency virus (HIV) disease	B20
Pneumocystosis	B59
Retrovirus as the cause of diseases classified elsewhere	B97.3
Post-transplant lymphoproliferative disorder (PTLD)	D47.Z1
Neutropenia (including agranulocytosis)	D70

© 2022 Montgomery MP et al. *JAMA Network Open.*

eTable 1. List of *ICD-10-CM* and Procedure Codes for Underlying Medical Conditions

Functional disorders of polymorphonuclear neutrophils	D71
Other disorders of white blood cells	D72
Diseases of spleen	D73
Other specified diseases with participation of lymphoreticular and reticulohistiocytic tissue	D76
Immunodeficiency with predominantly antibody defects	D80
Combined immunodeficiencies	D81
Immunodeficiency associated with other major defects	D82
Common variable immunodeficiency	D83
Other immunodeficiencies	D84
Other disorders involving the immune mechanism, not elsewhere classified	D89
Rheumatoid arthritis with rheumatoid factor	M05
Other rheumatoid arthritis	M06
Enteropathic arthropathies	M07
Juvenile arthritis	M08
Polyarteritis nodosa and related conditions	M30
Other necrotizing vasculopathies	M31
Systemic lupus erythematosus (SLE)	M32
Dermatopolymyositis	M33
Systemic sclerosis (scleroderma)	M34
Sicca syndrome (Sjögren)	M35.0
Systemic involvement of connective tissue, unspecified	M35.9
Congenital absence and malformations of spleen	Q89.0
Asymptomatic human immunodeficiency virus (HIV) infection status	Z21
Encounter for aftercare following organ transplant	Z48.2
Encounter for antineoplastic radiation therapy	Z51.0
Encounter for antineoplastic chemotherapy and immunotherapy	Z51.1
Transplanted organ and tissue status	Z94
Long term (current) use of steroids	Z79.5
Transplant complication	T86
**Substance use disorder**	
Alcohol abuse, uncomplicated	F10.10
Alcohol abuse with intoxication	F10.12
Alcohol abuse with alcohol-induced mood disorder	F10.14

© 2022 Montgomery MP et al. *JAMA Network Open.*

eTable 1. List of *ICD-10-CM* and Procedure Codes for Underlying Medical Conditions

Alcohol abuse with alcohol-induced psychotic disorder	F10.15
Alcohol abuse with other alcohol-induced disorders	F10.18
Alcohol abuse with unspecified alcohol-induced disorder	F10.19
Alcohol dependence, uncomplicated	F10.20
Alcohol dependence, in remission	F10.21
Alcohol dependence with intoxication	F10.22
Alcohol dependence with withdrawal	F10.23
Alcohol dependence with alcohol-induced mood disorder	F10.24
Alcohol dependence with alcohol-induced psychotic disorder	F10.25
Alcohol dependence with alcohol-induced persisting amnestic disorder	F10.26
Alcohol dependence with alcohol-induced persisting dementia	F10.27
Alcohol dependence with other alcohol-induced disorders	F10.28
Alcohol dependence with unspecified alcohol-induced disorder	F10.29
Alcohol use, unspecified with intoxication	F10.92
Alcohol use, unspecified with withdrawal	F10.93
Alcohol use, unspecified with alcohol-induced mood disorder	F10.94
Alcohol use, unspecified with alcohol-induced psychotic disorder	F10.95
Alcohol use, unspecified with alcohol-induced persisting amnestic disorder	F10.96
Alcohol use, unspecified with alcohol-induced persisting dementia	F10.97
Alcohol use, unspecified with other alcohol-induced disorders	F10.98
Alcohol use, unspecified with unspecified alcohol-induced disorder	F10.99
Opioid abuse, uncomplicated	F11.10
Opioid abuse with intoxication	F11.12
Opioid abuse with opioid-induced mood disorder	F11.14
Opioid abuse with opioid-induced psychotic disorder	F11.15
Opioid abuse with other opioid-induced disorder	F11.18
Opioid abuse with unspecified opioid-induced disorder	F11.19
Opioid dependence	F11.2
Opioid use, unspecified	F11.9
Cannabis abuse, uncomplicated	F12.10
Cannabis abuse with intoxication	F12.12
Cannabis abuse with psychotic disorder	F12.15
Cannabis abuse with other cannabis-induced disorder	F12.18

© 2022 Montgomery MP et al. *JAMA Network Open.*

eTable 1. List of *ICD-10-CM* and Procedure Codes for Underlying Medical Conditions

Cannabis abuse with unspecified cannabis-induced disorder	F12.19
Cannabis dependence, uncomplicated	F12.20
Cannabis dependence, in remission	F12.21
Cannabis dependence with intoxication	F12.22
Cannabis dependence with withdrawal	F12.23
Cannabis dependence with psychotic disorder	F12.25
Cannabis dependence with other cannabis-induced disorder	F12.28
Cannabis dependence with unspecified cannabis-induced disorder	F12.29
Cannabis use, unspecified, uncomplicated	F12.90
Cannabis use, unspecified with intoxication	F12.92
Cannabis use, unspecified with withdrawal	F12.93
Cannabis use, unspecified with psychotic disorder	F12.95
Cannabis use, unspecified with other cannabis-induced disorder	F12.98
Cannabis use, unspecified with unspecified cannabis-induced disorder	F12.99
Sedative, hypnotic or anxiolytic abuse, uncomplicated	F13.10
Sedative, hypnotic or anxiolytic abuse with intoxication	F13.12
Sedative, hypnotic or anxiolytic abuse with sedative, hypnotic or anxiolytic-induced mood disorder	F13.14
Sedative, hypnotic or anxiolytic abuse with sedative, hypnotic or anxiolytic-induced psychotic disorder	F13.15
Sedative, hypnotic or anxiolytic abuse with other sedative, hypnotic or anxiolytic-induced disorders	F13.18
Sedative, hypnotic or anxiolytic abuse with unspecified sedative, hypnotic or anxiolytic-induced disorder	F13.19
Sedative, hypnotic or anxiolytic-related dependence	F13.2
Sedative, hypnotic or anxiolytic-related use, unspecified	F13.9
Cocaine abuse, uncomplicated	F14.10
Cocaine abuse with intoxication	F14.12
Cocaine abuse with cocaine-induced mood disorder	F14.14
Cocaine abuse with cocaine-induced psychotic disorder	F14.15
Cocaine abuse with other cocaine-induced disorder	F14.18
Cocaine abuse with unspecified cocaine-induced disorder	F14.19
Cocaine dependence, uncomplicated	F14.20
Cocaine dependence, in remission	F14.21
Cocaine dependence with intoxication, uncomplicated	F14.220
Cocaine dependence with intoxication delirium	F14.221
Cocaine dependence with intoxication with perceptual disturbance	F14.222

© 2022 Montgomery MP et al. *JAMA Network Open.*

eTable 1. List of *ICD-10-CM* and Procedure Codes for Underlying Medical Conditions

Cocaine dependence with intoxication, unspecified	F14.229
Cocaine dependence with withdrawal	F14.23
Cocaine dependence with cocaine-induced mood disorder	F14.24
Cocaine dependence with cocaine-induced psychotic disorder	F14.25
Cocaine dependence with other cocaine-induced disorder	F14.28
Cocaine dependence with unspecified cocaine-induced disorder	F14.29
Cocaine use, unspecified, uncomplicated	F14.90
Cocaine use, unspecified with intoxication	F14.92
Cocaine use, unspecified with withdrawal	F14.93
Cocaine use, unspecified with cocaine-induced mood disorder	F14.94
Cocaine use, unspecified with cocaine-induced psychotic disorder	F14.95
Cocaine use, unspecified with other specified cocaine-induced disorder	F14.98
Cocaine use, unspecified with unspecified cocaine-induced disorder	F14.99
Other stimulant abuse, uncomplicated	F15.10
Other stimulant abuse with intoxication	F15.12
Other stimulant abuse with stimulant-induced mood disorder	F15.14
Other stimulant abuse with stimulant-induced psychotic disorder	F15.15
Other stimulant abuse with other stimulant-induced disorder	F15.18
Other stimulant abuse with unspecified stimulant-induced disorder	F15.19
Other stimulant dependence	F15.2
Other stimulant use, unspecified	F15.9
Hallucinogen abuse, uncomplicated	F16.10
Hallucinogen abuse with intoxication	F16.12
Hallucinogen abuse with hallucinogen-induced mood disorder	F16.14
Hallucinogen abuse with hallucinogen-induced psychotic disorder	F16.15
Hallucinogen abuse with other hallucinogen-induced disorder	F16.18
Hallucinogen abuse with unspecified hallucinogen-induced disorder	F16.19
Hallucinogen dependence	F16.2
Hallucinogen use, unspecified	F16.9
Inhalant abuse, uncomplicated	F18.10
Inhalant abuse with intoxication	F18.12
Inhalant abuse with inhalant-induced mood disorder	F18.14
Inhalant abuse with inhalant-induced psychotic disorder	F18.15

© 2022 Montgomery MP et al. *JAMA Network Open.*

eTable 1. List of *ICD-10-CM* and Procedure Codes for Underlying Medical Conditions

Inhalant abuse with inhalant-induced dementia	F18.17
Inhalant abuse with other inhalant-induced disorders	F18.18
Inhalant abuse with unspecified inhalant-induced disorder	F18.19
Inhalant dependence	F18.2
Inhalant use, unspecified	F18.9
Other psychoactive substance abuse, uncomplicated	F19.10
Other psychoactive substance abuse with intoxication	F19.12
Other psychoactive substance abuse with psychoactive substance-induced mood disorder	F19.14
Other psychoactive substance abuse with psychoactive substance-induced psychotic disorder	F19.15
Other psychoactive substance abuse with psychoactive substance-induced persisting amnestic disorder	F19.16
Other psychoactive substance abuse with psychoactive substance-induced persisting dementia	F19.17
Other psychoactive substance abuse with other psychoactive substance-induced disorders	F19.18
Other psychoactive substance abuse with unspecified psychoactive substance-induced disorder	F19.19
Other psychoactive substance dependence, uncomplicated	F19.20
Other psychoactive substance dependence, in remission	F19.21
Other psychoactive substance dependence with intoxication	F19.22
Other psychoactive substance dependence with withdrawal	F19.23
Other psychoactive substance dependence with psychoactive substance-induced mood disorder	F19.24
Other psychoactive substance dependence with psychoactive substance-induced psychotic disorder	F19.25
Other psychoactive substance dependence with psychoactive substance-induced persisting amnestic disorder	F19.26
Other psychoactive substance dependence with psychoactive substance-induced persisting dementia	F19.27
Other psychoactive substance dependence with other psychoactive substance-induced disorders	F19.28
Other psychoactive substance dependence with unspecified psychoactive substance-induced disorder	F19.29
Other psychoactive substance use, unspecified	F19.9
**Tobacco use and smoking**	
Tobacco use	Z72.0
Personal history of nicotine dependence	Z87.891
Nicotine dependence	F17
Tobacco use disorder complicating pregnancy	O99.33
Toxic effect of tobacco and nicotine	T65.2
Procedure codes for smoking and tobacco use cessation	CPT†: 99406, 99407, G0375, G0376, G0436, G0437, G8402, G8403, G8453, G8454, S4990, S4991,

© 2022 Montgomery MP et al. *JAMA Network Open.*

eTable 1. List of *ICD-10-CM* and Procedure Codes for Underlying Medical Conditions

	S4995, S9075, S9453, 4000F, 4001F
**Serious mental illness**	
Schizophrenia	F20
Schizoaffective disorders	F25
Bipolar disorders	F31
Severe major depression	F32.2, F32.3, F33.2, F33.3
**Disability**	
Barth syndrome	E78.71
Smith-Lemli-Opitz syndrome	E78.72
Mild intellectual disabilities	F70
Moderate intellectual disabilities	F71
Severe intellectual disabilities	F72
Profound intellectual disabilities	F73
Other intellectual disabilities	F78
Unspecified intellectual disabilities	F79
Specific developmental disorders of speech and language	F80
Specific developmental disorders of scholastic skills	F81
Specific developmental disorder of motor function	F82
Autistic disorder	F84.0
Other childhood disintegrative disorder	F84.3
Asperger's syndrome	F84.5
Other pervasive developmental disorders	F84.8
Pervasive developmental disorder, unspecified	F84.9
Tropical spastic paraplegia	G04.1
Hereditary spastic paraplegia	G11.4
Mild cognitive impairment, so stated	G31.84
Hemiplegia and hemiparesis	G81
Paraplegia (paraparesis) and quadriplegia (quadriparesis)	G82
Other paralytic syndromes	G83
Visual disturbances	H53
Blindness and low vision	H54
Conductive hearing loss, unilateral with unrestricted hearing on the contralateral side	H90.1

© 2022 Montgomery MP et al. *JAMA Network Open.*

eTable 1. List of *ICD-10-CM* and Procedure Codes for Underlying Medical Conditions

Sensorineural hearing loss, bilateral	H90.3
Sensorineural hearing loss, unilateral with unrestricted hearing on the contralateral side	H90.4
Unspecified sensorineural hearing loss	H90.5
Mixed conductive and sensorineural hearing loss, bilateral	H90.6
Mixed conductive and sensorineural hearing loss, unilateral with unrestricted hearing on the contralateral side	H90.7
Mixed conductive and sensorineural hearing loss, unspecified	H90.8
Sensorineural hearing loss, unilateral, with restricted hearing on the contralateral side	H90.A2
Mixed conductive and sensorineural hearing loss, unilateral with restricted hearing on the contralateral side	H90.A3
Ototoxic hearing loss	H91.0
Deaf nonspeaking, not elsewhere classified	H91.3
Other specified hearing loss	H91.8
Unspecified hearing loss	H91.9
Central auditory processing disorder	H93.25
Monoplegia of upper limb following nontraumatic subarachnoid hemorrhage	I69.03
Monoplegia of lower limb following nontraumatic subarachnoid hemorrhage	I69.04
Hemiplegia and hemiparesis following nontraumatic subarachnoid hemorrhage	I69.05
Other paralytic syndrome following nontraumatic subarachnoid hemorrhage	I69.06
Monoplegia of upper limb following nontraumatic intracerebral hemorrhage	I69.13
Monoplegia of lower limb following nontraumatic intracerebral hemorrhage	I69.14
Hemiplegia and hemiparesis following nontraumatic intracerebral hemorrhage	I69.15
Other paralytic syndrome following nontraumatic intracerebral hemorrhage	I69.16
Monoplegia of upper limb following other nontraumatic intracranial hemorrhage	I69.23
Monoplegia of lower limb following other nontraumatic intracranial hemorrhage	I69.24
Hemiplegia and hemiparesis following other nontraumatic intracranial hemorrhage	I69.25
Other paralytic syndrome following other nontraumatic intracranial hemorrhage	I69.26
Monoplegia of upper limb following cerebral infarction	I69.33
Monoplegia of lower limb following cerebral infarction	I69.34
Hemiplegia and hemiparesis following cerebral infarction	I69.35
Other paralytic syndrome following cerebral infarction	I69.36
Monoplegia of upper limb following other cerebrovascular disease	I69.83
Monoplegia of lower limb following other cerebrovascular disease	I69.84
Hemiplegia and hemiparesis following other cerebrovascular disease	I69.85
Other paralytic syndrome following other cerebrovascular disease	I69.86

© 2022 Montgomery MP et al. *JAMA Network Open.*

eTable 1. List of *ICD-10-CM* and Procedure Codes for Underlying Medical Conditions

Monoplegia of upper limb following unspecified cerebrovascular disease	I69.93
Monoplegia of lower limb following unspecified cerebrovascular disease	I69.94
Hemiplegia and hemiparesis following unspecified cerebrovascular disease	I69.95
Other paralytic syndrome following unspecified cerebrovascular disease	I69.96
Newborn affected by maternal use of alcohol	P04.3
Fetal alcohol syndrome (dysmorphic)	Q86.0
Congenital malformation syndromes predominantly associated with short stature	Q87.1
Congenital malformation syndromes predominantly involving limbs	Q87.2
Congenital malformation syndromes involving early overgrowth	Q87.3
Other congenital malformation syndromes with other skeletal changes	Q87.5
Alport syndrome	Q87.81
Other specified congenital malformation syndromes, not elsewhere classified	Q87.89
Multiple congenital malformations, not elsewhere classified	Q89.7
Other specified congenital malformations	Q89.8
Down syndrome	Q90
Trisomy 18 and Trisomy 13	Q91
Other trisomies and partial trisomies of the autosomes, not elsewhere classified	Q92
Whole chromosome monosomy, nonmosaicism (meiotic nondisjunction)	Q93.0
Whole chromosome monosomy, mosaicism (mitotic nondisjunction)	Q93.1
Chromosome replaced with ring, dicentric or isochromosome	Q93.2
Deletion of short arm of chromosome 4	Q93.3
Deletion of short arm of chromosome 5	Q93.4
Other deletions of part of a chromosome	Q93.5
Deletions with other complex rearrangements	Q93.7
Velo-cardio-facial syndrome	Q93.81
Other microdeletions	Q93.88
Other deletions from the autosomes	Q93.89
Deletion from autosomes, unspecified	Q93.9
Balanced autosomal rearrangement in abnormal individual	Q95.2
Balanced sex/autosomal rearrangement in abnormal individual	Q95.3
Fragile X chromosome	Q99.2
Abnormalities of gait and mobility	R26
Dyslexia and alexia	R48.0

© 2022 Montgomery MP et al. *JAMA Network Open.*

eTable 1. List of *ICD-10-CM* and Procedure Codes for Underlying Medical Conditions

Limitation of activities due to disability	Z73.6
Dual sensory impairment	Z73.82
Other reduced mobility	Z74.09
* These conditions were only considered underlying medical conditions if they were reported in an encounter that preceded the index COVID-19 encounter	
†CPT = current procedural terminology	

© 2022 Montgomery MP et al. *JAMA Network Open.*

eTable 2. List of *ICD-10-CM* and Procedure Codes for Acute Complications

**Respiratory**		
	Pneumonia	
	Viral pneumonia	J12
	Streptococcus pneumoniae pneumonia	J13
	Hemophilus influenzae pneumonia	J14
	Other bacterial pneumonia	J15
	Pneumonia due to other specified organism	J16
	Pneumonia in infectious diseases classified elsewhere	J17
	Pneumonia, unspecified organism	J18
	Abscess of lung with pneumonia	J85.1
	Influenza due to identified novel influenza A virus with pneumonia	J09.X1
	Influenza due to other identified influenza virus with pneumonia	J10.0
	Influenza due to unidentified influenza virus with pneumonia	J11.0
	Respiratory failure	
	Acute respiratory failure	J96.0
	Acute and chronic respiratory failure	J96.2
	Respiratory arrest	R09.2
	Acute respiratory distress syndrome	
	Acute respiratory distress syndrome	J80
	Asthma exacerbation	
	Mild intermittent asthma with acute exacerbation	J45.21
	Mild intermittent asthma with status asthmaticus	J45.22
	Mild persistent asthma with acute exacerbation	J45.31
	Mild persistent asthma with status asthmaticus	J45.32
	Moderate persistent asthma with acute exacerbation	J45.41
	Moderate persistent asthma with status asthmaticus	J45.42
	Severe persistent asthma with acute exacerbation	J45.51
	Severe persistent asthma with status asthmaticus	J45.52
	Unspecified asthma with acute exacerbation	J45.901
	Unspecified asthma with status asthmaticus	J45.902
	COPD exacerbation	
	COPD exacerbation	J44.1
	Pneumothorax	

© 2022 Montgomery MP et al. *JAMA Network Open.*

eTable 2. List of *ICD-10-CM* and Procedure Codes for Acute Complications

	Pneumothorax	J93
	Acute upper respiratory infections	
	Acute upper respiratory infections	J00-J06
	Mediastinitis	
	Mediastinitis	J98.51
	Other Acute Lower Respiratory Tract Disease	
	Acute bronchitis	J20
	Acute bronchiolitis	J21
	Unspecified acute lower respiratory infection	J22
	COPD with acute lower respiratory infection	J44.0
	Bronchiectasis with acute lower respiratory infection	J47.1
	Bronchiectasis with acute exacerbation	J47.9
	Gangrene and necrosis of lung	J85.0
	Abscess of lung without pneumonia	J85.2
	Abscess of mediastinum	J85.3
	Pyothorax	J86
	Pyothorax with fistula	J86.0
	Pyothorax without fistula	J86.9
	Pulmonary collapse	
	Atelectasis	J98.11
	Other pulmonary collapse	J98.19
**Cardiac**		
	Acute myocardial infarction or unstable angina	
	Unstable angina	I20.0
	Acute myocardial infarction	I21
	Subsequent ST elevation and non-ST elevation myocardial infarction	I22
	Other acute and subacute forms of ischemic heart disease	I24
	Acute Congestive Heart Failure	
	Acute systolic heart failure	I50.21
	Acute on chronic systolic heart failure	I50.23
	Acute diastolic heart failure	I50.31
	Acute on chronic diastolic heart failure	I50.33
	Acute combined systolic and diastolic heart failure	I50.41

© 2022 Montgomery MP et al. *JAMA Network Open.*

eTable 2. List of *ICD-10-CM* and Procedure Codes for Acute Complications

	Acute on chronic combined systolic and diastolic heart failure	I50.43
	Acute right heart failure	I50.811
	Acute on chronic right heart failure	I50.813
	Cardiogenic Shock	
	Cardiogenic Shock	R57.0
	Hypertensive Crisis	
	Hypertensive Crisis	I16
	Acute Myocarditis	
	Acute myocarditis	I40
	Influenza due to other identified influenza virus with myocarditis	J10.82
	Influenza due to unidentified influenza virus with myocarditis	J11.82
	Acute Pericarditis	
	Acute Pericarditis	I30
	Cardiac Tamponade	
	Cardiac Tamponade	I31.4
**Hematologic/vascular**		
	Deep vein thrombosis	
	Acute embolism and thrombosis of superior vena cava	I82.210
	Acute embolism and thrombosis of other thoracic veins	I82.290
	Acute embolism and thrombosis of inferior vena cava	I82.220
	Acute embolism and thrombosis of unspecified deep veins of lower extremity	I82.4
	Acute embolism and thrombosis of veins of upper extremity	I82.6
	Acute embolism and thrombosis of axillary vein	I82.A1
	Acute embolism and thrombosis of subclavian vein	I82.B1
	Acute embolism and thrombosis of internal jugular vein	I82.C1
	Pulmonary embolism	
	Pulmonary embolism	I26
	Disseminated intravascular coagulation	
	Disseminated intravascular coagulation	D65
	Hemaphagocytic Syndrome	
	Hemophagocytic lymphohistiocytosis	D76.1
	Hemophagocytic syndrome, infection-associated	D76.2
	Immune Thrombocytopenic Purpura	

© 2022 Montgomery MP et al. *JAMA Network Open.*

eTable 2. List of *ICD-10-CM* and Procedure Codes for Acute Complications

	Immune Thrombocytopenic Purpura	D69.3
**Neurologic**		
	Cerebral Ischemia/Infarction	
	Cerebral Infarction	I63
	Acute cerebrovascular insufficiency	I67.81
	Cerebral Ischemia	I67.82
	Transient cerebral ischemic attacks and related syndromes	G45
	Intracranial Hemorrhage	
	Intracranial Hemorrhage	I60, I61, I62
	Acute Disseminated Encephalitis and Encephalomyelitis	
	Acute Disseminated Encephalitis and Encephalomyelitis	G04.0
	Demyelinating Disease	
	Demyelinating Disease	G36
	Encephalitis	
	Encephalitis	G04-G05
	Guillain-Barre Syndrome	
	Guillain-Barre Syndrome	G61.0
	Meningitis	
	Meningitis	G00, G01, G02, G03
**Endocrine**		
	Diabetic Ketoacidosis	
	Diabetes mellitus due to underlying condition with ketoacidosis	E08.1
	Drug or chemical induced diabetes mellitus with ketoacidosis	E09.1
	Type 1 diabetes mellitus with ketoacidosis	E10.1
	Type 2 diabetes mellitus with ketoacidosis	E11.1
	Other specified diabetes mellitus with ketoacidosis	E13.1
	Hyperglycemic Hyperosmolar Syndrome	
	Diabetes mellitus due to underlying condition with hyperosmolarity	E08.0
	Drug or chemical induced diabetes mellitus with hyperosmolarity	E09.0
	Type 2 diabetes mellitus with hyperosmolarity	E11.0
	Other specified diabetes mellitus with hyperosmolarity	E13.0
	Thyrotoxicosis	
	Thyrotoxicosis	E05

© 2022 Montgomery MP et al. *JAMA Network Open.*

eTable 2. List of *ICD-10-CM* and Procedure Codes for Acute Complications

**Gastrointestinal**		
	Acute hepatitis and liver failure	
	Acute and subacute hepatic failure	K72.0
	Acute hepatitis A	B15
	Acute hepatitis B	B16
	Other acute viral hepatitis	B17
	Unspecified viral hepatitis with hepatic coma	B19.0
	Unspecified viral hepatitis B with hepatic coma	B19.11
	Unspecified viral hepatitis C with hepatic coma	B19.21
	Acute pancreatitis	
	Acute pancreatitis	K85
**Renal**		
	Acute Kidney Failure	
	Acute Kidney Failure	N17
	Dialysis	
	Encounter for care involving renal dialysis	Z49
	Dependence on renal dialysis	Z99.2
	Hypotension of hemodialysis	I95.3
	Patient's noncompliance with renal dialysis	Z91.15
	Hemodialysis procedure	CPT*: 90935, 90937
	Hemodialysis access flow study to determine blood flow in grafts and arteriovenous fistulae by an indicator method	CPT: 90940
	Dialysis procedure other than hemodialysis (e.g., peritoneal dialysis, hemofiltration, or other continuous renal replacement therapies)	CPT: 90945, 90947
	Other dialysis procedures	CPT: 90999
	Hemodialysis plan docd	CPT: 0505F
	Hemodialysis via catheter	CPT: 4054F
	Insertion of cannula for hemodialysis, other purpose (separate procedure)	CPT: 36800, 36810, 36815
**Sepsis**		
	Streptococcal sepsis	A40
	Other sepsis	A41
	Symptoms and signs specifically associated with systemic inflammation and infection	R65
*CPT = current procedural terminology		

© 2022 Montgomery MP et al. *JAMA Network Open.*

eTable 3. Full Multivariable Results for COVID-19 Hospitalization Outcomes Among People Experiencing Incarceration and People Experiencing Homelessness, United States, April 2020–June 2021

	**ICU Admission**					
	**Admitted**		**Unadjusted**		**Fully adjusted**	
	n	%	RR†	95% CI	RR†	95% CI
Population						
PEI*	683	31.5	0.99	0.80, 1.23	0.95	0.80, 1.13
PEH	1922	31.6	1.00	0.90, 1.10	0.92	0.85, 1.01
General population	197683	31.7	Reference		Reference	
Age						
Continuous (10-year increment)			1.05	1.04, 1.06	1.06	1.05, 1.07
Sex						
Male	108750	33.5	1.13	1.11, 1.14	1.12	1.10, 1.13
Female	91538	29.7	Reference		Reference	
Race and ethnicity						
Asian, non-Hispanic	4786	29.4	0.92	0.79, 1.07	0.90	0.80, 1.01
Black, non-Hispanic	34202	30.2	0.94	0.86, 1.04	1.01	0.93, 1.10
White, non-Hispanic	109656	32.0	Reference		Reference	
Hispanic	35660	34.0	1.06	0.92, 1.23	1.04	0.90, 1.20
Other race, non-Hispanic	11257	29.3	0.92	0.76, 1.11	0.95	0.82, 1.11
Unknown	4727	28.1	0.88	0.73, 1.06	0.96	0.84, 1.11
Geographic divisions (1st encounter)						
Northeast	22740	20.0	0.60	0.43, 0.83	0.60	0.43, 0.83
Midwest	35817	27.1	0.81	0.65, 1.00	0.80	0.65, 0.99
South	99203	33.5	Reference		Reference	
West	42528	46.8	1.40	1.15, 1.69	1.40	1.15, 1.69
Rural/Urban (1st encounter)						
Rural	20799	27.2	0.84	0.68, 1.05	0.81	0.66, 1.00
Urban	179489	32.3	Reference		Reference	
Other medical comorbidities‖						
Serious mental illness	8189	30.8	0.97	0.93, 1.02	1.04	1.00, 1.07
No serious mental illness	192099	31.7	Reference		Reference	
Disability						
Disability	28830	33.2	1.06	1.01, 1.10	1.03	1.00, 1.07
No disability	171458	31.4	Reference		Reference	
Wave						
April 1, 2020 to May 31, 2020	18073	25.8	Reference		Reference	
June 1, 2020 to August 31, 2020	35311	36.6	1.42	1.23, 1.63	1.13	1.04, 1.24
September 1, 2020 to June 30, 2021	146904	31.5	1.22	1.09, 1.36	1.05	0.96, 1.14

Abbreviations: CI, confidence interval; ICU, intensive care unit; PEH, people experiencing homelessness; PEI, people experiencing incarceration; RR, risk ratio; IRR, incident rate ratio

* Patients with ICD-10-CM codes for PEI and PEH are included in PEI cohort

† Log-binomial model, risk ratio reported

‡ Alternative revised Poisson model, risk ratio reported

§ Zero-truncated negative binomial model, incident rate ratio reported

‖ Not mutually exclusive, estimated separately

eTable 3. Full Multivariable Results for COVID-19 Hospitalization Outcomes Among People Experiencing Incarceration and People Experiencing Homelessness, United States, April 2020–June 2021

	**Invasive Mechanical Ventilation**					
	**Ventilated**		**Unadjusted**		**Fully adjusted**	
	No.	%	RR†	95% CI	RR†	95% CI
Population						
PEI*	401	18.9	1.33	1.19, 1.48	1.16	1.04, 1.30
PEH	606	10.0	0.70	0.63, 0.77	0.64	0.58, 0.70
General population	88897	14.2	Reference		Reference	
Age						
Continuous (10-year increment)			1.06	1.05, 1.07	1.07	1.06, 1.08
Sex						
Male	54250	16.7	1.44	1.42, 1.46	1.44	1.42, 1.46
Female	35663	11.6	Reference		Reference	
Race and ethnicity						
Asian, non-Hispanic	2649	16.3	1.22	1.15, 1.30	1.22	1.16, 1.30
Black, non-Hispanic	16085	14.2	1.06	1.02, 1.11	1.13	1.09, 1.18
White, non-Hispanic	45803	13.4	Reference		Reference	
Hispanic	15713	15.0	1.12	1.05, 1.20	1.19	1.11, 1.27
Other race, non-Hispanic	6752	17.6	1.32	1.24, 1.40	1.33	1.24, 1.42
Unknown	2911	17.3	1.29	1.20, 1.40	1.34	1.24, 1.44
Geographic divisions (1st encounter)						
Northeast	15795	13.9	1.01	0.92, 1.10	0.91	0.83, 0.99
Midwest	18566	14.0	1.01	0.93, 1.11	1.01	0.92, 1.10
South	40914	13.8	Reference		Reference	
West	14638	16.1	1.16	1.05, 1.29	1.16	1.04, 1.29
Rural/Urban (1st encounter)						
Rural	10210	13.4	0.93	0.84, 1.04	0.97	0.87, 1.08
Urban	79703	14.3	Reference		Reference	
Other medical comorbidities‖						
Serious mental illness	4060	15.3	1.08	1.04, 1.12	1.14	1.10, 1.19
No serious mental illness	85853	14.2	Reference		Reference	
Disability						
Disability	13897	16.0	1.15	1.11, 1.19	1.09	1.06, 1.12
No disability	76016	13.9	Reference		Reference	
Wave						
April 1, 2020 to May 31, 2020	12542	17.9	Reference		Reference	
June 1, 2020 to August 31, 2020	13789	14.3	0.80	0.75, 0.84	0.79	0.76, 0.83
September 1, 2020 to June 30, 2021	63582	13.6	0.76	0.73, 0.79	0.77	0.74, 0.80

Abbreviations: CI, confidence interval; ICU, intensive care unit; PEH, people experiencing homelessness; PEI, people experiencing incarceration; RR, risk ratio; IRR, incident rate ratio

* Patients with ICD-10-CM codes for PEI and PEH are included in PEI cohort

† Log-binomial model, risk ratio reported

‡ Alternative revised Poisson model, risk ratio reported

§ Zero-truncated negative binomial model, incident rate ratio reported

‖ Not mutually exclusive, estimated separately

© 2022 Montgomery MP et al. *JAMA Network Open.*

eTable 3. Full Multivariable Results for COVID-19 Hospitalization Outcomes Among People Experiencing Incarceration and People Experiencing Homelessness, United States, April 2020–June 2021

	**In-Hospital Mortality**					
	**Died**		**Unadjusted**		**Fully adjusted**	
	No.	%	RR‡	95% CI	RR‡	95% CI
Population						
PEI*	308	14.2	1.05	0.89, 1.23	1.28	1.11, 1.47
PEH	330	5.4	0.40	0.35, 0.45	0.53	0.47, 0.59
General population	84725	13.6	Reference		Reference	
Age						
Continuous (10-year increment)			1.43	1.42, 1.44	1.45	1.44, 1.47
Sex						
Male	50454	15.5	1.37	1.35, 1.39	1.41	1.39, 1.43
Female	34909	11.3	Reference		Reference	
Race and ethnicity						
Asian, non-Hispanic	2222	13.6	0.98	0.92, 1.05	1.09	1.03, 1.16
Black, non-Hispanic	13743	12.1	0.87	0.84, 0.91	1.11	1.07, 1.15
White, non-Hispanic	47563	13.9	Reference		Reference	
Hispanic	13106	12.5	0.90	0.84, 0.97	1.23	1.14, 1.32
Other race, non-Hispanic	6156	16.0	1.16	1.07, 1.26	1.31	1.23, 1.40
Unknown	2573	15.3	1.10	1.03, 1.18	1.30	1.22, 1.37
Geographic divisions (1st encounter)						
Northeast	18345	16.2	1.28	1.18, 1.38	1.08	1.00, 1.16
Midwest	16554	12.5	0.99	0.92, 1.06	0.93	0.87, 1.00
South	37446	12.7	Reference		Reference	
West	13018	14.3	1.13	1.04, 1.23	1.18	1.08, 1.28
Rural/Urban (1st encounter)						
Rural	9785	12.8	0.94	0.86, 1.03	0.95	0.86, 1.04
Urban	75578	13.6	Reference		Reference	
Other medical comorbidities‖						
Serious mental illness	3330	12.5	0.93	0.89, 0.97	1.12	1.08, 1.17
No serious mental illness	82033	13.5	Reference		Reference	
Disability						
Disability	15529	17.9	1.40	1.36, 1.43	1.08	1.06, 1.11
No disability	69834	12.8	Reference		Reference	
Wave						
April 1, 2020 to May 31, 2020	13057	18.7	Reference		Reference	
June 1, 2020 to August 31, 2020	11863	12.3	0.66	0.61, 0.71	0.75	0.70, 0.79
September 1, 2020 to June 30, 2021	60443	13.0	0.69	0.66, 0.73	0.74	0.71, 0.77

Abbreviations: CI, confidence interval; ICU, intensive care unit; PEH, people experiencing homelessness; PEI, people experiencing incarceration; RR, risk ratio; IRR, incident rate ratio

* Patients with ICD-10-CM codes for PEI and PEH are included in PEI cohort

† Log-binomial model, risk ratio reported

‡ Alternative revised Poisson model, risk ratio reported

§ Zero-truncated negative binomial model, incident rate ratio reported

‖ Not mutually exclusive, estimated separately

© 2022 Montgomery MP et al. *JAMA Network Open.*

eTable 3. Full Multivariable Results for COVID-19 Hospitalization Outcomes Among People Experiencing Incarceration and People Experiencing Homelessness, United States, April 2020–June 2021

	30-Day Readmission for COVID-19					
	Readmitted		Unadjusted		Fully adjusted	
	No.	%	RR‡	95% CI	RR‡	95% CI
Population						
PEI*	128	5.9	1.29	1.06, 1.57	1.45	1.18, 1.78
PEH	519	8.5	1.87	1.71, 2.04	2.10	1.92, 2.30
General population	28493	4.6	Reference		Reference	
Age						
Continuous (10-year increment)			1.21	1.20, 1.22	1.20	1.19, 1.22
Sex						
Male	15884	4.9	1.14	1.11, 1.16	1.13	1.11, 1.16
Female	13256	4.3	Reference		Reference	
Race and ethnicity						
Asian, non-Hispanic	648	4.0	0.79	0.71, 0.87	0.90	0.81, 0.99
Black, non-Hispanic	5317	4.7	0.93	0.89, 0.97	1.05	1.01, 1.09
White, non-Hispanic	17344	5.1	Reference		Reference	
Hispanic	3843	3.7	0.72	0.69, 0.76	0.89	0.85, 0.94
Other race, non-Hispanic	1457	3.8	0.75	0.70, 0.81	0.86	0.80, 0.92
Unknown	531	3.2	0.62	0.57, 0.69	0.71	0.65, 0.78
Geographic divisions (1st encounter)						
Northeast	5231	4.6	1.01	0.92, 1.10	1.02	0.94, 1.10
Midwest	6519	4.9	1.08	1.01, 1.15	1.05	0.99, 1.11
South	13551	4.6	Reference		Reference	
West	3839	4.2	0.92	0.86, 0.99	0.97	0.90, 1.03
Rural/Urban (1st encounter)						
Rural	3755	4.9	1.08	1.01, 1.15	1.02	0.96, 1.09
Urban	25385	4.6	Reference		Reference	
Other medical comorbidities‖						
Serious mental illness	1595	6.0	1.32	1.25, 1.39	1.33	1.26, 1.40
No serious mental illness	27545	4.5	Reference		Reference	
Disability						
Disability	5659	6.5	1.51	1.46, 1.57	1.28	1.24, 1.33
No disability	23481	4.3	Reference		Reference	
Wave						
April 1, 2020 to May 31, 2020	3016	4.3	Reference		Reference	
June 1, 2020 to August 31, 2020	4879	5.1	1.18	1.10, 1.25	1.27	1.20, 1.35
September 1, 2020 to June 30, 2021	21245	4.6	1.06	1.00, 1.12	1.07	1.01, 1.13

Abbreviations: CI, confidence interval; ICU, intensive care unit; PEH, people experiencing homelessness; PEI, people experiencing incarceration; RR, risk ratio; IRR, incident rate ratio

* Patients with ICD-10-CM codes for PEI and PEH are included in PEI cohort

† Log-binomial model, risk ratio reported

‡ Alternative revised Poisson model, risk ratio reported

§ Zero-truncated negative binomial model, incident rate ratio reported

‖ Not mutually exclusive, estimated separately

© 2022 Montgomery MP et al. *JAMA Network Open.*

eTable 3. Full Multivariable Results for COVID-19 Hospitalization Outcomes Among People Experiencing Incarceration and People Experiencing Homelessness, United States, April 2020–June 2021

	**Length of Stay**					
	**Days**		**Unadjusted**		**Fully adjusted**	
	Mean	Standard deviation	IRR§	95% CI	IRR§	95% CI
Population						
PEI*	9.03	10.13	1.11	1.06, 1.16	1.11	1.06, 1.16
PEH	10.68	25.56	1.23	1.20, 1.26	1.24	1.20, 1.27
General population	8.36	9.68	Reference		Reference	
Age						
Continuous (10-year increment)			1.10	1.10, 1.10	1.10	1.10, 1.11
Sex						
Male	8.93	10.67	1.16	1.15, 1.16	1.16	1.16, 1.17
Female	7.81	9.10	Reference		Reference	
Race and ethnicity						
Asian, non-Hispanic	9.06	10.76	1.03	1.01, 1.05	1.09	1.07, 1.11
Black, non-Hispanic	8.53	10.13	1.00	0.99, 1.01	1.06	1.05, 1.07
White, non-Hispanic	8.07	8.96	Reference		Reference	
Hispanic	8.82	11.20	1.04	1.03, 1.05	1.15	1.14, 1.16
Other race, non-Hispanic	9.11	12.92	1.04	1.03, 1.05	1.11	1.10, 1.12
Unknown	8.91	10.83	1.05	1.03, 1.07	1.11	1.09, 1.13
Geographic divisions (1st encounter)						
Northeast	9.11	11.81	1.11	1.03, 1.20	1.02	0.95, 1.09
Midwest	7.61	8.39	0.89	0.84, 0.95	0.89	0.84, 0.94
South	8.34	9.64	Reference		Reference	
West	8.76	10.42	0.98	0.91, 1.05	0.98	0.92, 1.05
Rural/Urban (1st encounter)						
Rural	7.28	7.77	0.72	0.69, 0.76	0.72	0.69, 0.76
Urban	8.54	10.21	Reference		Reference	
Other medical comorbidities‖						
Serious mental illness	9.76	12.05	1.19	1.18, 1.21	1.23	1.21, 1.25
No serious mental illness	8.33	9.85	Reference		Reference	
Disability						
Disability	10.11	12.00	1.28	1.27, 1.29	1.22	1.21, 1.22
No disability	8.11	9.56	Reference		Reference	
Wave						
April 1, 2020 to May 31, 2020	9.36	11.03	Reference		Reference	
June 1, 2020 to August 31, 2020	8.71	10.66	0.93	0.92, 0.94	0.95	0.94, 0.96
September 1, 2020 to June 30, 2021	8.18	9.62	0.89	0.89, 0.90	0.91	0.90, 0.92

Abbreviations: CI, confidence interval; ICU, intensive care unit; PEH, people experiencing homelessness; PEI, people experiencing incarceration; RR, risk ratio; IRR, incident rate ratio

* Patients with ICD-10-CM codes for PEI and PEH are included in PEI cohort

† Log-binomial model, risk ratio reported

‡ Alternative revised Poisson model, risk ratio reported

§ Zero-truncated negative binomial model, incident rate ratio reported

‖ Not mutually exclusive, estimated separately

© 2022 Montgomery MP et al. *JAMA Network Open.*

eTable 4. Sensitivity Analyses for COVID-19 Hospitalization Outcomes for People Experiencing Incarceration and People Experiencing Homelessness, United States, April 2020–June 2021

	PEI defined by ICD-10-CM codes PEI n=1021; PEH n=6,236		PEI defined by admission code PEI n=1,530; PEH n=6,191		Controlling for Payer Source PEI n=2,170; PEH n=6,088		Controlling for Underlying Medical Conditions PEI n=2,170; PEH n=6,088	
	RR or IRR	95% CI	RR or IRR	95% CI	RR or IRR	95% CI	RR or IRR	95% CI
ICU Admission†								
PEI*	1.10	0.94, 1.28	0.87	0.69, 1.09	0.97	0.82, 1.15	0.94	0.79, 1.11
PEH	0.92	0.84, 1.00	0.92	0.85, 1.00	0.95	0.88, 1.03	0.89	0.81, 0.96
Invasive Mechanical Ventilation†								
PEI*	1.43	1.25, 1.63	0.93	0.81, 1.08	1.17	1.04, 1.31	1.14	1.03, 1.26
PEH	0.64	0.58, 0.70	0.63	0.58, 0.69	0.62	0.57, 0.68	0.55	0.50, 0.61
In-Hospital Mortality‡								
PEI*	1.51	1.27, 1.79	1.07	0.88, 1.30	1.32	1.15, 1.52	1.26	1.09, 1.45
PEH	0.52	0.46, 0.58	0.52	0.46, 0.59	0.51	0.45, 0.57	0.47	0.42, 0.53
30-Day Readmission for COVID-19‡								
PEI*	1.48	1.13, 1.94	1.60	1.27, 2.00	1.53	1.25, 1.86	1.44	1.18, 1.77
PEH	2.13	1.96, 2.33	2.11	1.93, 2.30	1.99	1.82, 2.18	1.98	1.81, 2.16
Length of Stay§								
PEI*	1.17	1.10, 1.25	1.04	0.99, 1.10	1.12	1.07, 1.17	1.13	1.09, 1.18
PEH	1.23	1.19, 1.26	1.23	1.20, 1.26	1.21	1.18, 1.25	1.14	1.11, 1.17

Abbreviations: CI, confidence interval; ICU, intensive care unit; PEH, people experiencing homelessness; PEI, people experiencing incarceration; RR, risk ratio; IRR, incident rate ratio

* Patients with ICD-10-CM codes for PEI and PEH are included in PEI cohort

† Log-binomial model, risk ratio reported

‡ Alternative revised Poisson model, risk ratio reported

§ Zero-truncated negative binomial model, incident rate ratio reported

© 2022 Montgomery MP et al. *JAMA Network Open*.
